# Research towards selective inhibition of the CLK3 kinase

**DOI:** 10.3762/bjoc.21.172

**Published:** 2025-10-24

**Authors:** Vinay Kumar Singh, Frédéric Justaud, Dabbugoddu Brahmaiah, Nangunoori Sampath Kumar, Blandine Baratte, Thomas Robert, Stéphane Bach, Chada Raji Reddy, Nicolas Levoin, René L Grée

**Affiliations:** 1 Univ Rennes, CNRS, ISCR (Institut des Sciences Chimiques de Rennes), UMR 6226, F-35000 Rennes, France; 2 D.N.(P.G.) College, Khudwadhar, Gulaothi, Bulandshahr, Uttar Pradesh-203408, India; 3 Chemveda Life Sciences India Pvt. Ltd., B-11/1, IDA Uppal, Hyderabad-500039, Telangana, India; 4 Sorbonne Université, CNRS, FR 2424, Plateforme de criblage KISSf (Kinase Inhibitor Specialized Screening facility), Station Biologique de Roscoff, CS 90074, 29688 Roscoff Cedex, France; 5 Sorbonne Université, CNRS, UMR 8227, Integrative Biology of Marine Models Laboratory (LBI2M), Station Biologique de Roscoff, CS 90074, 29688 Roscoff Cedex, France; 6 CSIR-Indian Institute of Chemical Technology, Uppal Road, Tarnaka, Hyderabad 500007, TS, India; 7 Bioprojet-Biotech, 4 rue du Chesnay Beauregard, BP 96205, 35762 Saint Grégoire, France

**Keywords:** cancer, CLK3, kinases, molecular modelling, quinazolines, triazoles

## Abstract

The cdc2-like kinases (CLKs), are a family of kinases that attracted recently the interest of scientists due to their significant biological roles, in particular in the regulation of the mRNA splicing process. Among the four isoforms of CLKs, CLK3 is the one for which the biological roles are less understood, in part because no selective inhibitor of this challenging kinase has been found to date. Based on structural analysis of the CLKs we have identified the lysine 241, present only in CLK3, as an attractive residue to design inhibitors with increased affinity towards this kinase as compared to the three other isoforms CLK1, CLK2, and CLK4. Based on this observation, we have been able to transform a molecule (**DB18**) previously established with a very low activity on CLK3 into a derivative **VS-77** which has now a significant affinity toward CLK3 (IC_50_ = 0.3 μM). Thus, **VS-77** appears as a new pan-inhibitor of the CLK family.

## Introduction

Human protein kinases are a family comprising nearly 535 phosphotransferases (called the human kinome) involved in specific signaling pathways which regulate cell functions (e.g., metabolism, cell cycle progression, cell adhesion, vascular function, and angiogenesis). Therefore, the dysregulation of protein kinase enzymatic activity, induced by genetic alterations as well as overexpression, is implicated in the pathogenesis of numerous deleterious diseases including nervous and inflammatory disorders as well as a number of malignancies [1–3]. Kinases are also known to be highly druggable by both allosteric and competitive inhibitors. As a consequence, protein kinases have become one of the most important drug targets: between a quarter to a third of the drug discovery efforts worldwide are focused on the discovery of new protein kinase inhibitors. More than 80 FDA-approved drugs that target about two dozen different protein kinases were discovered during the last 25 years and more than 400 orally effective protein kinase inhibitors are in clinical trials worldwide [4–5]. However, and despite this high interest, the function in human biology of approximately one third of the kinase members is poorly understood [6]. These enzymes are classified as “dark kinases” because of the lack of functional annotations and high-quality molecular probes for functional investigations [7–8]. Thus, new studies are still required for the discovery of selective inhibitors for each of these kinases in order to better clarify their mechanisms of action and their roles in living systems. It is important to classify the kinases regarding the level of knowledge that scientists have on their physiological and pathological roles. Oprea et al. defined a knowledge-based protein classification that led to the definition of four groups: from the more studied T_clin_ (*clinic*, with approved drug on the market), T_chem_ (*chemistry*, known to bind to small molecules with high potency), T_bio_ (*biology*, kinases that have notably gene ontology leaf term annotations associated with disease and cellular role but lack associations with bioactive molecules) to the less explored T_dark_ (*dark* genome, that do not meet any of the criteria for the other classes, see ref [9] for details).

Among the T_chem_ are the members of the cdc2-like kinases (CLKs) family. They have attracted the interest of scientists due to their biological roles in many areas and significant involvement in human diseases [10–12]. In particular, these CLKs are involved in the regulation of mRNAs splicing with important consequences especially in cancer [13]. There are four isoforms of CLKs (CLK1, CLK2, CLK3, and CLK4) whose structures have been clearly established and are available in PDB.

Among the CLKs family, CLK3 appears presently as the less studied, although it has been implicated in cancer as well as in other unrelated diseases like malaria [14–15]. Further, it has been proposed to play also a role in the formation of the central nervous system [16]. The lack of knowledge around CLK3 is likely due to the fact that no potent and selective inhibitor of this specific kinase has been reported, to the best of our knowledge. Among the T_chem_ kinase inhibitors [9], only four molecules demonstrated significant enzymatic inhibition of CLK3 (from 6.5 nM to 110 nM, Table 1, [10]): SM08502, T-025, T3, and CX-4945.

**Table 1:** Structures and in vitro activities of known CLK3 inhibitors. The IC_50_ values are given in nM. For T-025**,** *K*_d_ values (in nM) are specified.

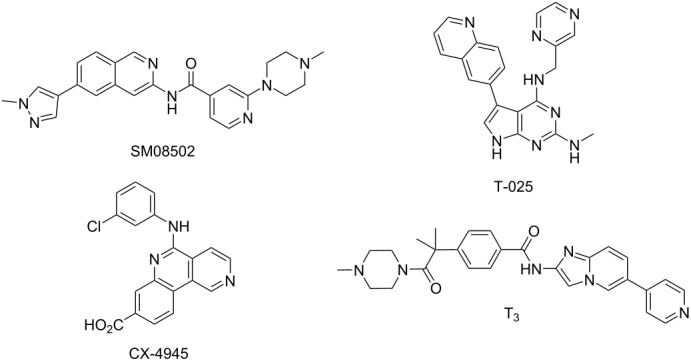								

	Human protein kinases							
Compound Id	CLK1	CLK2	CLK3	CLK4	DYRK1A	DYRK1B	DYRK2	Refs

SM08502	8	1	22	1	1	1	3	[17]
T-025^a^	4.8	0.096	6.5	0.61	0.074	1.5	32	[18]
T3	0.67	15	110	ND	260	230	ND	[19]
CX-4945	3.3	2.9	67	23	14	ND	ND	[20–21]
DB18	0.011	0.027	1.280	0.020	>100	ND	ND	[24]

^a^*K*_d_ values.

However, these derivatives remain still less potent on CLK3 than on the other CLKs. Therefore, they cannot be qualified as bona fide chemical probes for this CLK3 kinase. Structural and biochemical studies have been undertaken to find more selective CLKs inhibitors. Particularly, a detailed study has indicated the important role of one residue located N-terminal to the DFG motif (called DFG-1). In this particular position, CLK3 has a small alanine, while the others have a bulkier valine. This offers a rationale to explain in particular why most, if not all, known inhibitors have lower affinity for CLK3 [22]. But because our goal is to enhance activity for CLK3, we cannot take advantage of this amino acid variation, and we had to investigate for other differences between CLKs.

Here, we report our strategy and first efforts towards this end, starting from compound **DB18** which is highly potent and selective inhibitor of CLK1, -2 and -4, with no activity on CLK3 [23–24], to obtain a new derivative **VS-77**, which reached submicromolar activity on CLK3. Extensive molecular modeling studies have been used here to design **VS-77** and to propose a model to explain the selectivity observed.

## Strategy and Design of the Targets

**Figure 1:**
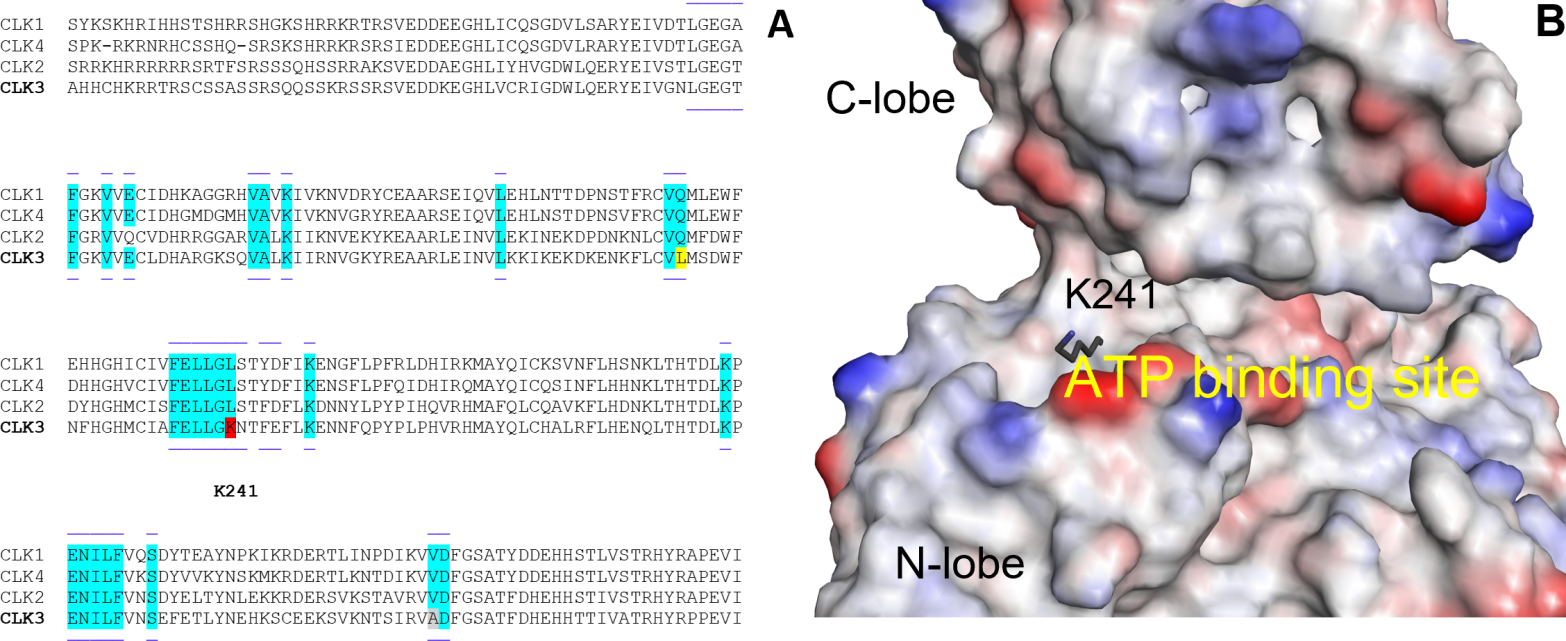
A) Sequence of amino acids in CLK3 as compared to the three other CLKs; B) Structure of CLK3 highlighting the lysine in position 241 (PDB ID: 2WU6 [25]).

Our study started by a detailed analysis of the sequence of amino acids in CLK3 compared to the three other CLKs. There are several differences but one appeared very significant (Figure 1A): CLK3 has a polar lysine in position 241 while the other CLKs have a nonpolar leucine. Further, examination of the structure of CLK3 shows that this key lysine 241 is very close to the entry of the ATP binding site (Figure 1B).

**Figure 2:**
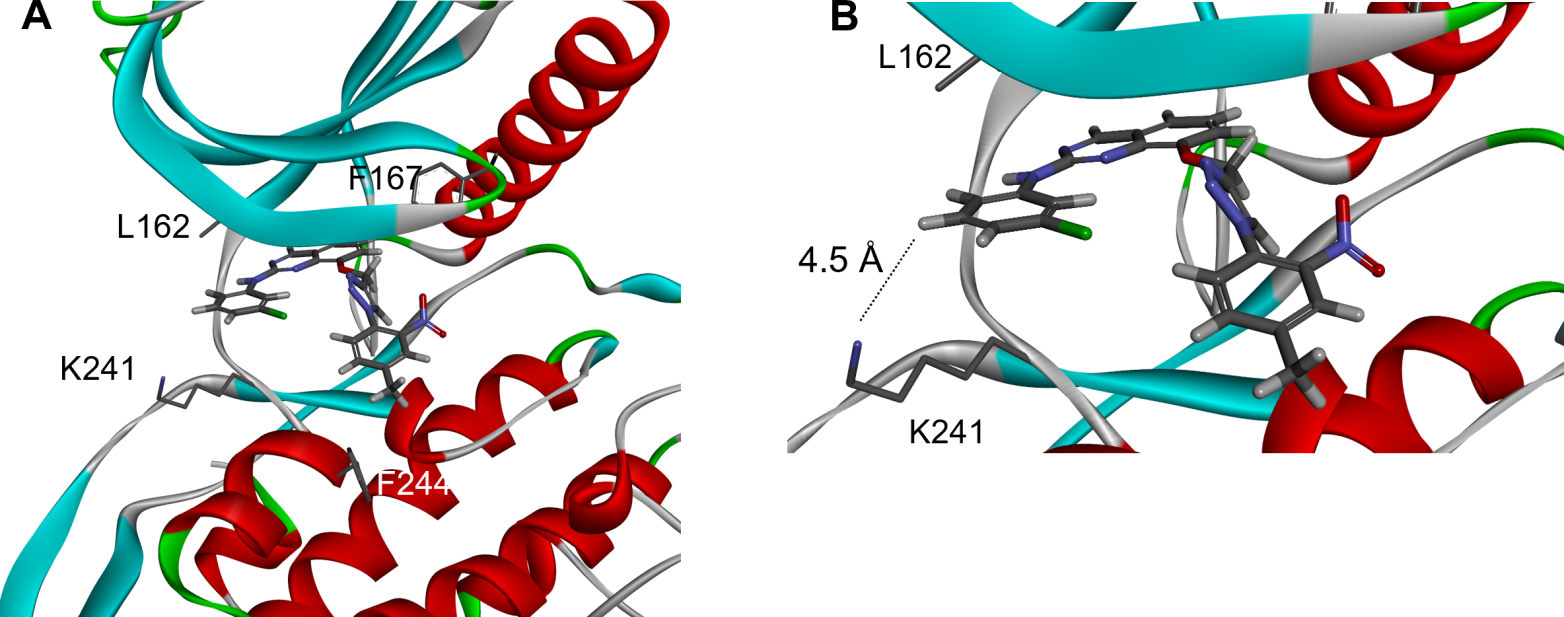
A) Docking of our previous inhibitor (**DB18**) in CLK3 and B) our working hypothesis.

Lysine 241 could be considered as an opportunity to design new molecules with an improved affinity to CLK3, by adding specific interactions with this amino acid bearing a primary amine in terminal position. Based on this, our strategy was to design new inhibitors with introduction, in their terminal part, of an acid group which could perform an extra hydrogen bond or a salt bridge with lysine 241 and therefore could be specific to CLK3. Towards this goal we started from our previously described **DB18** [23–24], a moderate inhibitor (IC_50_ = 1.28 μM) docked into CLK3 (Figure 2A), and we proposed to introduce, through an appropriate linker, an acid group close to this lysine 241 (Figure 2B).

**Figure 3:**
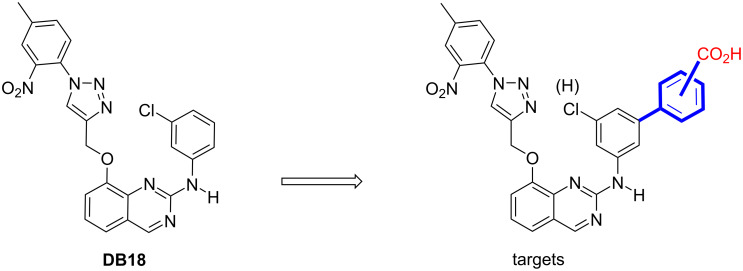
Design of our target molecules.

Preliminary studies indicated that a simple aromatic group could be very appropriate as a linker between the core of the inhibitor and the acidic function. The acid could be placed in *meta* or *para* positions taking into account the flexible backbone of lysine 241 (Figure 3). Further, in case the binding of these new targets would require a little more flexibility around the basic skeleton, we decided to prepare also the same molecules with hydrogen instead of the chlorine in *meta* position of the anilino group. Our previous work on **DB18** suggested indeed that the chlorine atom is implicated in intramolecular halogen–π interaction, ending in a conformational constraint and ligand rigidity [24].

## Results and Discussion

### Chemical syntheses

**Scheme 1:**
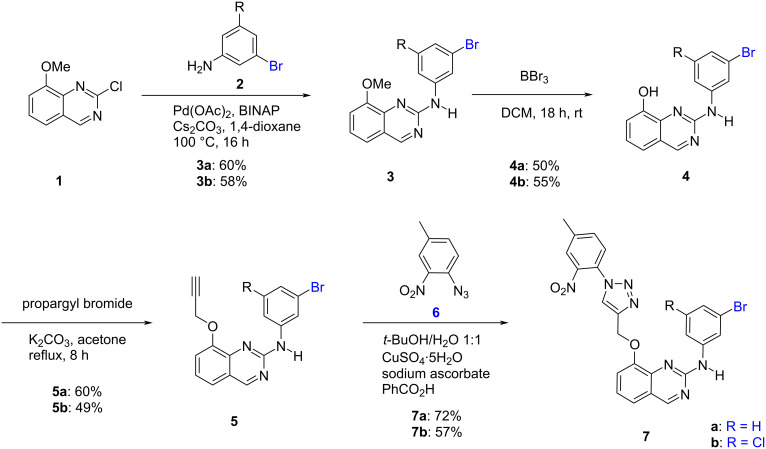
Synthesis of the intermediate anilino-2-quinazolines **7a** and **7b**.

The synthetic strategy is very similar to the one previously developed for the preparation of **DB18** and designed analogues (Scheme 1) [23–24]. It starts from known quinazoline **1** which, on Buchwald–Hartwig-type reaction with 3-bromoaniline (**2a**), gave anilinoquinazoline **3a**. Deprotection of the methoxy group by BBr_3_ gave phenol **4a** which was propargylated to intermediate **5a**. A final click-type reaction [27–31] with azide **6** gave the first target intermediate **7a**. The second key intermediate **7b** was prepared in a very similar manner, but starting from 3-bromo-5-chloroaniline (**2b**).

**Scheme 2:**
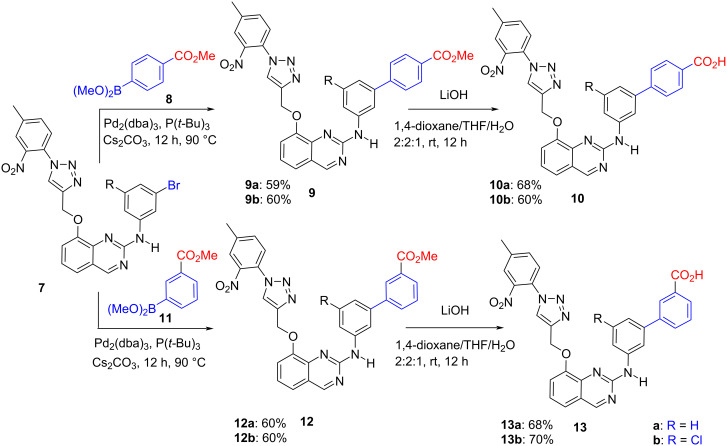
Synthesis of the targeted anilino-2-quinazolines **10** and **13**.

Next, we prepared the final targets by Suzuki-type reactions using the aromatic bromides **7**. As indicated before, the acid function designed to interact with the lysine 241 has been introduced both in *para* and *meta* positions of the aromatic linker. Further we have prepared two series of molecules: the ones without chlorine in *meta* position on this group (series **a**) and the others which kept this chlorine, like in **DB18** (series **b**). These syntheses are reported in Scheme 2. A Suzuki-type Pd-catalyzed coupling of **7a** with 4-(methoxycarbonyl)phenylboronic acid dimethyl ester (**8**) gave **9a** which, after saponification, afforded the first target compound **10a**. The second molecule **10b** was obtained in the same manner starting from **7b** with the chlorine in *meta* position of the aromatic group. Then, by a similar approach, the targets **13a** and **13b** were prepared by now using as the coupling reagent the 3-(methoxycarbonyl)phenylboronic acid ester **11**. All derivatives have spectral and analytical data in agreement with the proposed structures (see experimental section and Supporting Information File 1).

### Kinase inhibition studies

**Figure 4:**
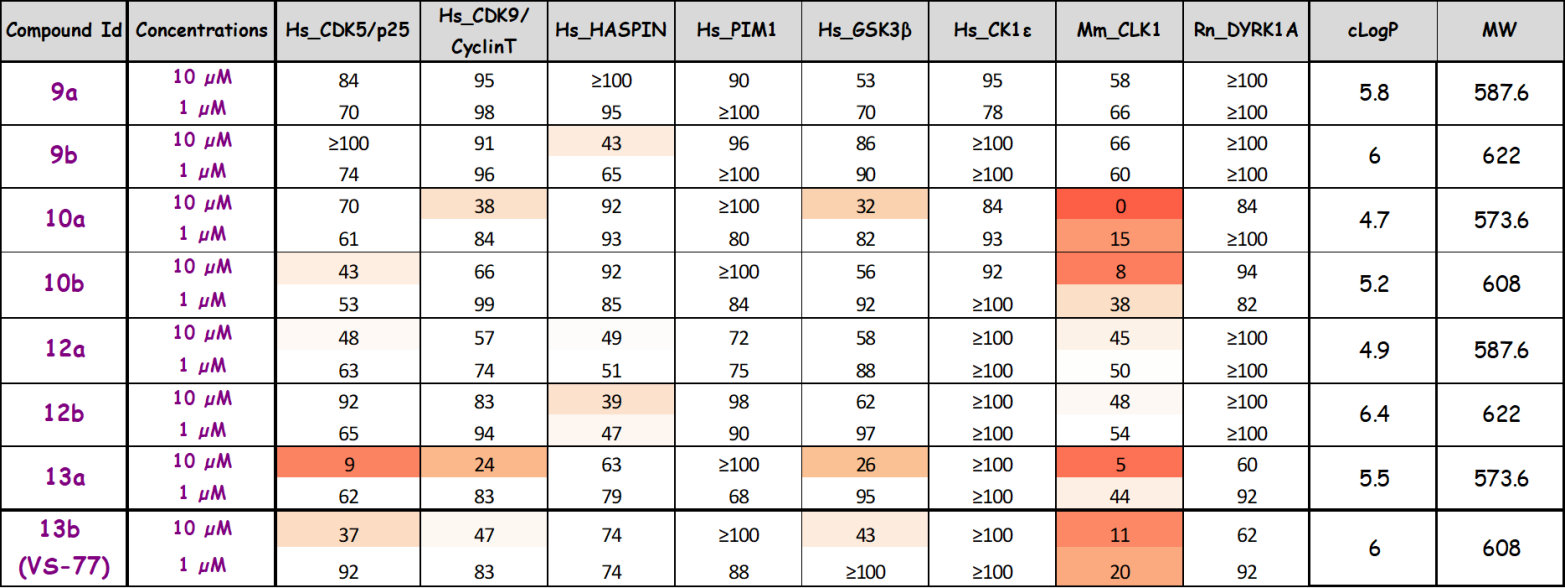
Primary evaluation of the inhibition of the new quinazolines against a short panel of mammalian kinases. Residual kinase activities are expressed in % of maximal activity, i.e., measured in the absence of inhibitor but with an equivalent dose of DMSO (solvent of the tested compounds). ATP concentration used in the kinase assays was 10 µM (values are means, *n* = 2). Kinases are from human origin (*Homo sapiens*) except CLK1 (from *Mus musculus)* and DYRK1A (*from Rattus norvegicus)*.

Our molecules have been submitted first to a primary screening against a panel of eight disease-related kinases: five members of the CMGC (for CDK, MAPK, GSK3, and CLK) group (CDK5/p25, CDK9/CyclinT, GSK3β, CLK1 and DYRK1A), one CAMK (calmodulin/calcium regulated kinase PIM1), one CK1 (the casein kinase CK1ε), and haspin as a typical kinase. The results obtained are reported in Figure 4. The four acids **10a**, **10b**, **13a**, and **13b** demonstrated a significant activity against CLK1 with very low to no action on the other selected kinases. On the other hand, the corresponding esters **9** and **12** were found to be not significantly active against the tested kinases. Indeed, we failed to observe a dose-dependent effect from 1 to 10 µM for these compounds (e.g., the same level of inhibition was observed when **12a** was tested at 1 or 10 µM against CLK1).

For the hit compounds that showed an inhibitory activity, we next determined their respective IC_50_ against the four mouse CLKs. The results are reported in Table 2 and in Supporting Information File 1. For comparison, we have also reported the values obtained earlier with our starting model compound **DB18** [23–24].

**Table 2:** IC_50_ data (in μM) for the inhibition of *Mm_CLK1-4* by the four quinazolines **10** and **13** plus **DB18**.^a^

Compound Id	Mm_CLK1 (Mm_CLK3/Mm_CLK1)	Mm_CLK2 (Mm_CLK3/Mm_CLK2)	Mm_CLK3	Mm_CLK4 (Mm_CLK3/Mm_CLK4)

**10a**	0.891 *(4.18)*	0.844 (*4.42*)	3.732	0.762 (*4.90*)
**10b**	0.213 *(4.04)*	0.118 (*7.29*)	0.861	0.145 (5.94)
**13a**	0.68 (*2.43*)	0.278 (*5.95*)	1.655	0.428 (*3.87*)
**13b** (**VS-77**)	0.127 (*2.38*)	0.061 (*4.97*)	0.303	0.062 (*4.89*)
**DB18** ^b^	0.011 (*116*)	0.027 (*47.4*)	1.28	0.02 (*64*)

^a^Kinase activities were measured by radiometric γ^33^P-ATP assay (Eurofins, UK) using 15 µM ATP. They were calculated from dose–response curves for which each point was measured in duplicate. ^b^Data taken from refs [23–24].

Four points arose from these SAR data: i) The four acid derivatives maintain a significant inhibition of the CLK1, CLK2, and CLK4, in the low micromolar range and even better (60 nM) for **13b** (= **VS-77**). ii) They show also inhibition of CLK3 in the same order of magnitude as for the three other CLKs from 0.3 μM for **VS-77** up to 3.7 μM for **10a**. iii) Although we did not obtain yet a selective CLK3 inhibitor, the acidic group brought a clear improvement in CLK3 affinity, either in *meta* or in *para* position (Figure 4). For the best compound **VS-77**, the inhibition of CLK3 (0.3 μM) is much closer to the other CLKs (IC_50_ from 0.06 μM to 0.12 μM) than in the case of **DB18**. iv) These results strongly support our hypothesis that the addition of the terminal acid significantly reinforces the interaction of our new molecules with CLK3, probably through a salt bridge with lysine 241. Logically, the corresponding esters are found to be inactive.

Finally, in order to complete our profiling of **VS-77**, we then measured its activity against the *DYRK 1A*, *DYRK1B* and *DYRK2* kinases and the results are reported in Table 3.

**Table 3:** IC_50_ data (in μM) for the inhibition of *Rn_*DYRK1A*, Hs_*DYRK1A*, Hs_*DYRK1B and *Hs_*DYRK2 by **VS-77** and **DB18**.^a^

Compound Id	*Rn_*DYRK1A	*Hs_*DYRK1A	*Hs_*DYRK1B	*Hs_*DYRK2

VS-77	14.76	5.31	4.6	11.69
DB18^b^	>100	>100	>100	>100

^a^Kinase activities were measured by radiometric γ^33^P-ATP assay (Eurofins, UK) using 15 µM ATP. They were calculated from dose-ponse curves for which each point was measured in duplicate. ^b^Data taken from refs [23–24].

To our surprise, and contrary to the results obtained earlier with **DB18**, the new compound **VS-77** exhibited low, but significant inhibition of these DYRK kinases. This point will be discussed in the molecular modeling part below.

### Molecular modeling studies

**Figure 5:**
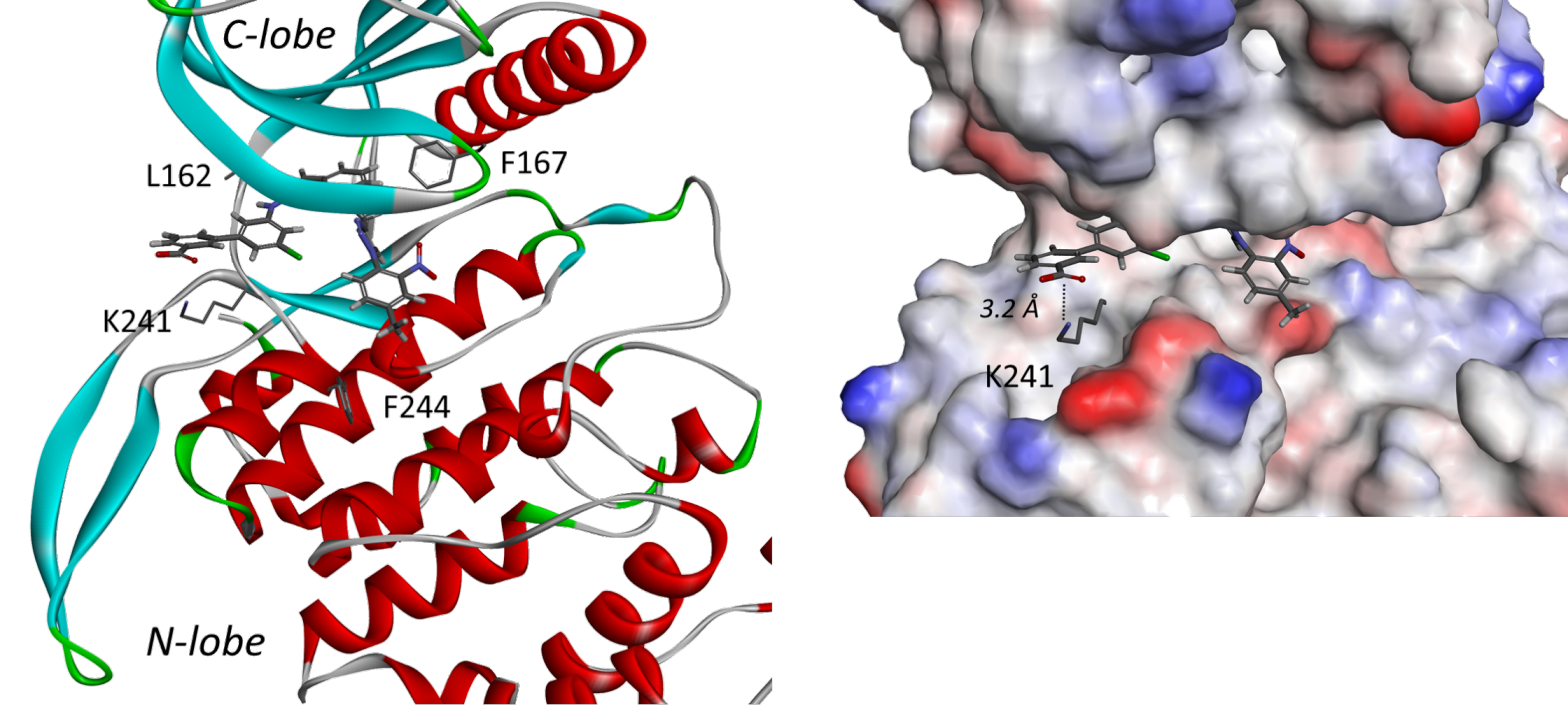
Docking of **VS-77** in CLK3.

First, molecular modeling experiments suggested that our initial hypothesis was correct: the most potent compound **VS-77** seems actually able to form a salt bridge with lysine 241, since the acid is located close to the amino group of this lysine (about 3 Å, Figure 5).

**Figure 6:**
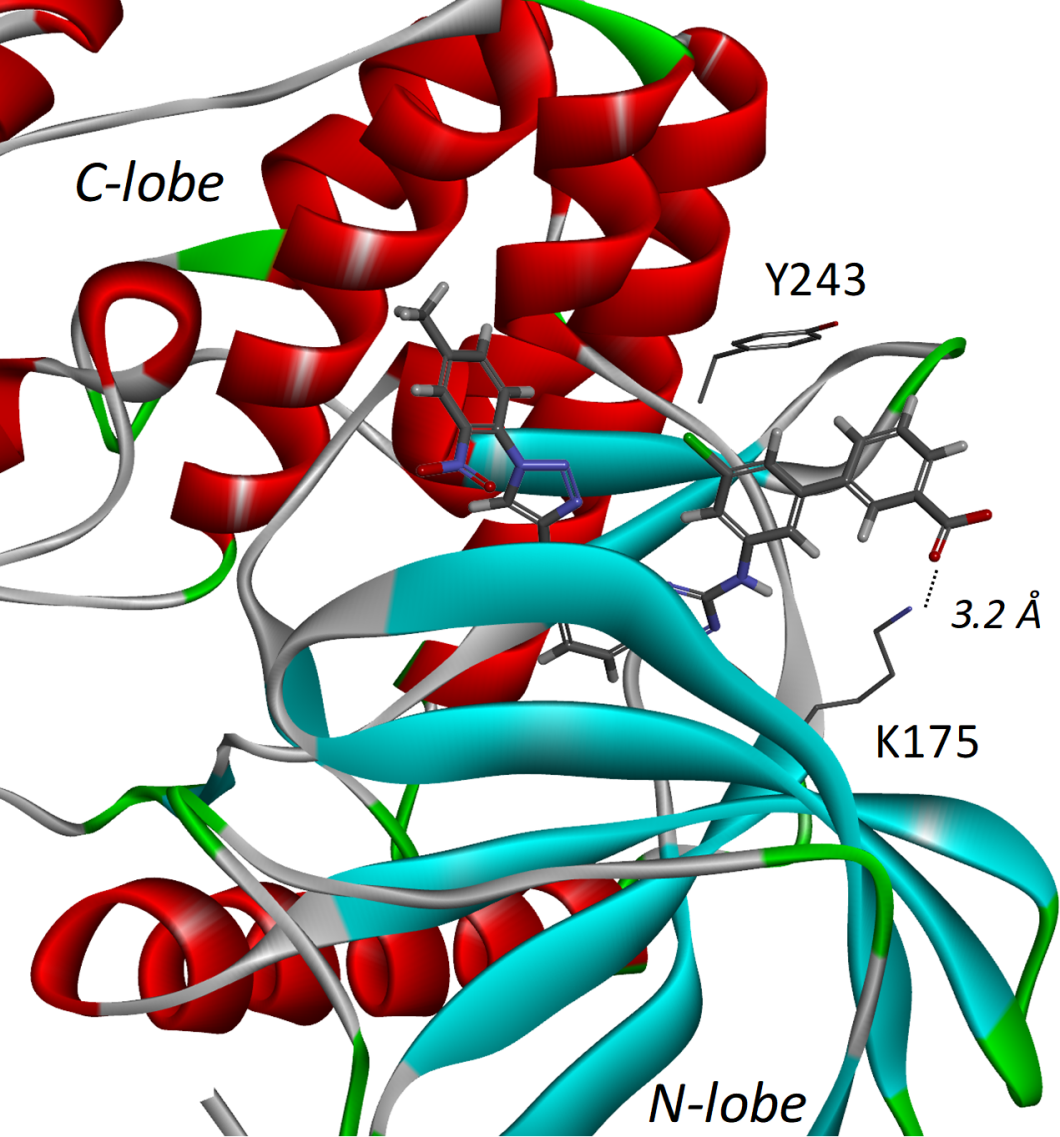
Docking of **VS-77** in *Hs_*DYRK1A (PDB ID: 8T2H [26]).

Second, molecular docking using DYRK1A crystal structure was used to understand the unexpected binding on the DYRK kinases (Figure 6). Surprisingly, another lysine (K175) is also located near the acidic group of **VS-77** (at 3.2 Å between the carbonyl carbon atom and nitrogen from amine). This residue is positioned on the opposite lobe of the protein, as compared to lysine 241 of CLK3 (N-lobe for DYRK1A vs C-lobe for CLK3), but the docking experiment showed that a 180° rotation of the benzoic acid allowed the inhibitor to interact with lysine 175. In addition to molecular simulations, this salt bridge is also evidenced by the better affinity for DYRK1A of the acidic molecules as compared to their ester analogues. Therefore, this new interaction could explain the higher affinity of **VS-77** toward *Hs_*DYRK1A, as compared to **DB-18**.

## Conclusion

In the present work, we disclosed a new strategy to improve the affinity of ligands towards the CLK3 kinase. It is based on the lysine 241 which is present only in CLK3, while the three others (CLK1, 2, and 4) have apolar leucine in this position. Thus, by grafting an extra benzoic acid on a molecule previously established as having low activity on CLK3 (**DB18**), we have been able to transform this compound into a derivative having a significant affinity toward this kinase (**VS-77,** IC_50_ = 0.3 μM). Further, this compound has kept good activities against the other CLKs, and therefore can be qualified as a new pan-inhibitor of the CLKs. Unexpectedly, this modification has also transformed **VS-77** into a weak inhibitor of the DYRK kinases, while **DB18** was inactive on later kinases. Analyses through molecular modeling allowed us to suggest that this change could be due, in the same way, to the interaction of the newly introduced acid with another basic residue, lysine 175 present in DYRK kinases. Nevertheless, by highlighting the role of the lysine 241, the present work paves the way towards the long-term goal of getting more potent and selective inhibitors of this understudied CLK3 kinase.

## Supporting Information

File 1 Detailed experimental procedures and spectral data, kinase inhibition studies, molecular modelling studies, analysis of dose-dependent effect of **VS-77** on the kinase activity of Mm_CLKs and copies of the ^1^H and ^13^C NMR spectra.

## Data Availability

All data that supports the findings of this study is available in the published article and/or the supporting information of this article.
