# Supplementary material for: Research towards selective inhibition of the CLK3 kinase
Source: Beilstein J Org Chem. 2025 Oct 24;21:2250–9. doi: 10.3762/bjoc.21.172 (PMC12557437; doi:10.3762/bjoc.21.172)
Supplement: File 1 — Detailed experimental procedures and spectral data, kinase inhibition studies, molecular modelling studies, analysis of dose-dependent effect of VS-77 on the kinase activity of Mm_CLKs and copies of the 1H and 13C NMR spectra. [file Beilstein_J_Org_Chem-21-2250-s001.pdf]

BEILSTEIN JOURNAL OF ORGANIC CHEMISTRY

Supporting Information

for

Research towards selective inhibition of the CLK3 kinase

Vinay Kumar Singh, Frédéric Justaud, Dabbugoddu Brahmaiah,
Nangunoori Sampath Kumar, Blandine Baratte, Thomas Robert, Stéphane Bach,
Chada Raji Reddy, Nicolas Levoin and René L. Grée

Beilstein J. Org. Chem. **2025,** *21,* 2250–2259. doi:10.3762/bjoc.21.172

Detailed experimental procedures and spectral data, kinase inhibition studies, molecular modelling studies, analysis of dose-dependent effect of VS-77 on the kinase activity of Mm_CLKs and copies of the ^{1}H and ^{13}C NMR spectra

License and Terms: This is a supporting information file under the terms of the Creative Commons Attribution License (https://creativecommons.org/licenses/by/4.0). Please note that the reuse, redistribution and reproduction in particular requires that the author(s) and source are credited and that individual graphics may be subject to special legal provisions.
The license is subject to the *Beilstein Journal of Organic Chemistry* terms and conditions: (https://www.beilstein-journals.org/bjoc/terms)

Table of contents

Figure S1: Analysis of dose-dependent effect of **VS-77** on the kinase activity of Mm_CLK1, Mm_CLK2, Mm_CLK3, Mm_CLK4 .... S3

Experimental S4

1- Chemical synthesis S4

2- Kinase inhibition studies S11

3- Molecular modelling studies S11

Figure S2: ^{1}H NMR spectrum of **3a** S12

Figure S3: ^{13}C NMR spectra of **3a** S13

Figure S4: ^{1}H NMR spectrum of **4a** S14

Figure S5: ^{1}H NMR spectrum of **5a** S15

Figure S6: ^{13}C NMR spectrum of **5a** S16

Figure S7: ^{1}H NMR spectrum of **7a** S17

Figure S8: ^{13}C NMR spectra of **7a** S18

Figure S9: ^{1}H NMR spectrum of **3b** S19

Figure S10: ^{13}C NMR spectrum of **3b** S20

Figure S11: ^{1}H NMR spectrum of **4b** S21

Figure S12: ^{13}C NMR spectra of **4b** S22

Figure S13: ^{1}H NMR spectrum of **5b** S23

Figure S14: ^{13}C NMR spectra of **5b** S24

Figure S15: ^{1}H NMR spectrum of **7b** S25

Figure S16: ^{13}C NMR spectrum of **7b** S26

Figure S17: Extension of ^{13}C NMR spectrum of **7b** S27

Figure S18: ^{1}H NMR spectrum of **9a** S28

Figure S19: ^{13}C NMR spectrum of **9a** S29

Figure S20: ^{1}H NMR spectrum of **10a** S30

Figure S21: ^{13}C NMR spectra of **10a** S31

Figure S22: ^{1}H NMR spectrum of **12a** S32

Figure S23: ^{13}C NMR spectrum of **12a** S33

Figure S24: ^{1}H NMR spectrum of **13a** S34

Figure S25: ^{13}C NMR spectrum of **13a** S35

Figure S26: ^{1}H NMR spectrum of **9b** S36

Figure S27: ^{13}C NMR spectra of **9b** S37

Figure S28: ^{1}H NMR spectrum of **10b** S38

Figure S29: ^{13}C NMR spectrum of **10b** S39

Figure S30: ^{1}H NMR spectrum of **12b** S40

Figure S31: ^{13}C NMR spectra of **12b** S41

Figure S32: ^{1}H NMR spectrum of **13b** S42

Figure S33: ^{13}C NMR spectrum of **13b** S43

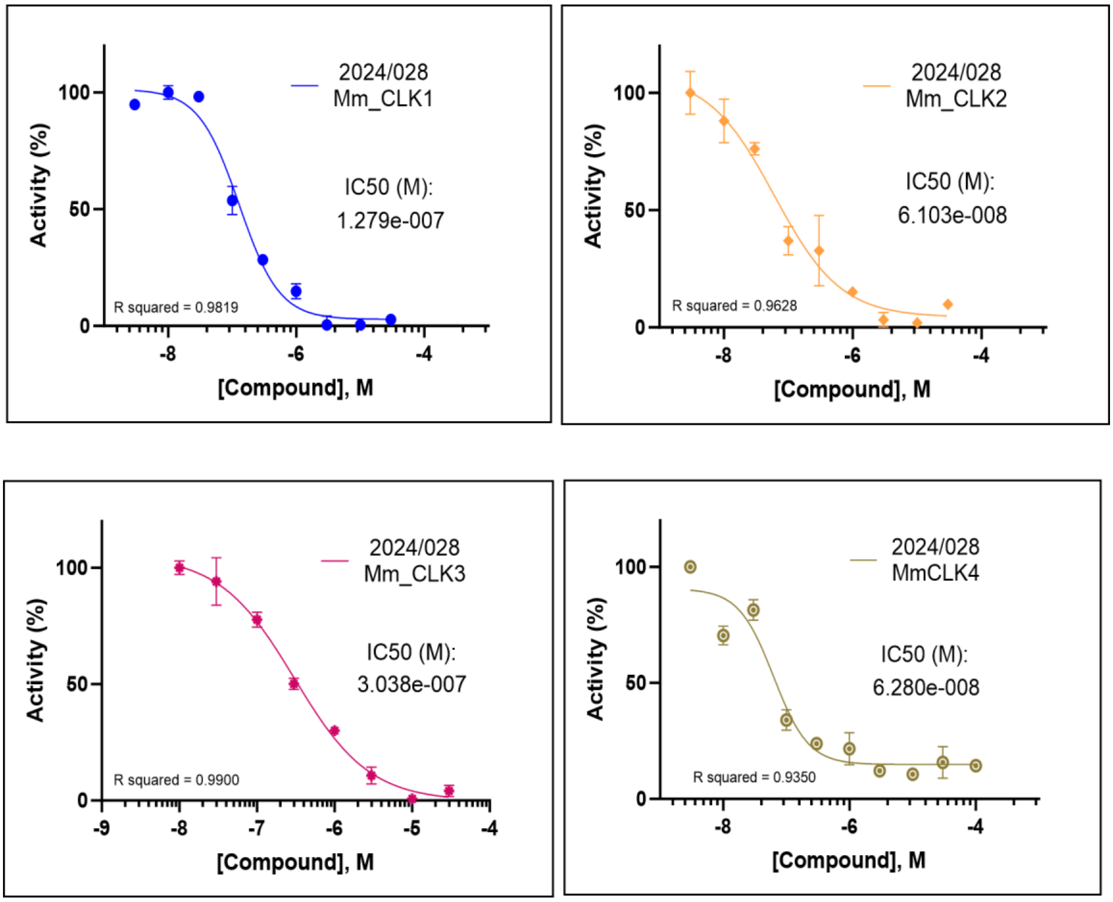

Figure S1: Analysis of dose-dependent effect of VS-77 on the kinase activity of Mm_CLK1, Mm_CLK2, Mm_CLK3, Mm_CLK4.

Experimental

1- Chemical synthesis

General information

All reactions were performed in heat gun-dried round-bottomed flasks under a dry argon or nitrogen atmosphere. Air and moisture-sensitive compounds were introduced via syringes or cannula, using standard inert atmosphere techniques. In addition, the gas stream was passed through a glass cylinder filled with P_2O_5 to remove any traces of residual moisture. Reactions were monitored by thin-layer chromatography (TLC) using E. Merck silica gel plates and components were visualized by illumination with short wavelength UV light and/or staining (ninhydrin or basic $KMnO_4$). All aldehydes were distilled right before use. All benzyl bromides and other reagents were used as they were received from commercial suppliers, unless otherwise noted. THF and Et_2O were dried over sodium-benzophenone and distilled prior to use. Anhydrous CH_2Cl_2 was prepared by refluxing in the presence of CaH_2 and distilled right before use unless otherwise noted.

^{1}H NMR spectra were recorded on Bruker spectrometers at 300 and 400 MHz, and ^{13}C NMR spectra at 75 and 100 MHz, in $CDCl_3$ or DMSO-d_6 using TMS (tetramethylsilane) as an internal standard. Multiplicity was tabulated using standard abbreviations: s for singlet, d for doublet, dd for doublet of doublets, t for triplet, q for quadruplet, ddd for doublet of doublets of doublets and m for multiplet (br means broad). When necessary, in particular in order to have better accuracy on small coupling constants, resolution in ^{1}H NMR was enhanced using Traficante. The synthetic strategy and the procedures for the preparation of intermediates **3**–**7** are similar to the one previously developed for the preparation of **DB18** and designed analogues [1,2]. All triazole compounds were purified by flash column chromatography on neutral alumina unless otherwise noted.

Sample availability: Samples of the compounds are available from authors.

Synthesis of N-(3-bromo-phenyl)-8-methoxyquinazolin-2-amine (3a)

To a stirred solution 2-chloro-8-methoxyquinazoline (**1**, 500 mg, 2.57 mmol) and 3-bromoaniline (**2a**, 570 g, 3.35 mmol) in 1,4-dioxane (2 mL) was added BINAP (150 mg, 0.257 mmol), $Pd(OAc)_2$ (28.8 mg, 0.05 mmol) and Cs_2CO_3 (219 mg, 6.75 mmol). Then, the

reaction mixture was evacuated under vacuum, back filled with Ar atmosphere and heated to 100 °C for 16 h. The reaction was monitored by TLC and after its completion, the reaction mixture was cooled to rt (room temperature) and then filtered through a celite pad. The filtrate was concentrated and to this crude material, ice and water were added and after extraction with ethyl acetate (2 × 100 mL), the combined organic extracts were washed with brine (50 mL). The organic layer was dried over anhydrous sodium sulfate and concentrated to dryness. The crude resulting product was purified by chromatography on silica gel eluting with 5–20% ethyl acetate in cyclohexane to give compound **3** (600 mg, 60% yield) as a yellow solid. ^{1}H NMR (300 MHz, $CDCl_3$) δ 9.14 (s, 1H), 8.25 (t, J = 1.9 Hz, 1H), 7.61 (dt, J = 7.5, 1.9 Hz, 1H), 7.46 (s, 1H), 7.39 (dd, J = 8.1, 1.4 Hz, 1H), 7.33 (d, J = 7.6 Hz, 1H), 7.30 – 7.13 (m, 4H), 4.10 (s, 3H).). 13C NMR (75 MHz, $CDCl_3$) δ 161.7, 156.3, 153.4, 143.6, 141.2, 130.1, 125.1, 124.0, 122.8, 121.7, 121.7, 119.02, 117.2, 112.6, 56.2. HRMS (ESI): m/z calcd for $C_{15}H_{13}N_3O^{79}Br$ [M + H]$^+$: 330.02365, found 330.0230 (1 ppm).

Synthesis of N-(3-bromo-5-chlorophenyl)-8-methoxyquinazolin-2-amine (3b)

Yield: 1.3 g (58%). ^{1}H NMR (400 MHz, DMSO-d_6) δ 10.35 (s, 1H), 9.36 (s, 1H), 8.38 (s, 1H), 8.27 (s, 1H), 7.52 (dd, *J*= 1.6 Hz, 7.6 Hz, 1H), 7.40-7.34 (m, 2H), 7.24 (t, *J*= 1.6 Hz, 1H), 3.99 (s, 3H). **^{13}C NMR** (75 MHz, DMSO) δ 162.6, 156.1, 153.5, 143.9, 142.7, 134.5, 124.8, 123.1, 122.5, 121.8, 119.6, 119.6, 117.1, 114.0, 56.5. HRMS (ESI): m/z calcd for $C_{15}H_{12}N_3O^{35}Cl^{79}Br$ [M + H]$^+$: 363.98468, found 363.9846 (0 ppm).

2-((3-Bromophenyl)amino)quinazolin-8-ol (4a)

To a stirred solution of *N*-(3-bromophenyl)-8-methoxyquinazolin-2-amine (**3a**, 500 mg, 1.51 mmol) in CH_2Cl_2 (1.5 mL) was added 2.9 mL of BBr_3 in CH_2Cl_2 (1.0 M) at 0 °C, and the reaction mixture was stirred at rt for 18 h. The reaction mixture was poured to a mixture of ice and water and stirred for 2 h at rt. The resulting solid was collected by filtration and washed with water (2 × 40 mL). The obtained pale-yellow solid was dried in a dessicator to give compound **4** (240 mg, 50% yield) as a yellow solid. ^{1}H NMR (300 MHz, $CDCl_3$) δ 9.13 (s, 1H), 8.03 – 7.96 (m, 1H), 7.59 (dt, *J* = 6.7, 2.4 Hz, 1H), 7.48 – 7.21 (m, 8H). HRMS (ESI): m/z calcd for $C_{14}H_{11}N\ O^{79}Br$ [M + H]$^+$: 316.008, found 316.0078 (1 ppm).

2-((3-Bromo-5-chlorophenyl)amino)quinazolin-8-ol (4b)

Same procedure starting from **3b** to obtain **4b**. Yield: 0.7 g (55%). ^{1}H NMR (400 MHz, DMSO-d_6) δ 10.23 (s, 1H), 9.99 (brs, 1H), 9.31 (s, 1H), 8.33 (t, *J*= 1.6 Hz, 1H), 8.28 (t, *J*= 2.0 Hz, 1H), 7.40 (dd, *J*= 1.2 Hz, 7.6 Hz, 1H), 7.29-7.21 (m, 3H). ^{13}C NMR (75 MHz, DMSO) δ 162.6, 155.7, 151.9, 143.9, 141.8, 134.5, 125.1, 123.1, 122.5, 122.2, 119.5, 118.3, 117.4, 117.1. HRMS (ESI): m/z calcd for $C_{14}H_{10}N_3O^{35}Cl^{79}Br$ [M + H]$^+$: 349.96903, found 349.9691 (0 ppm)

N-(3-Bromophenyl)-8-(prop-2-yn-1-yloxy)quinazolin-2-amine (5a)

To a stirred solution of 2-((3-bromophenyl)amino)quinazolin-8-ol (100 mg, 0.31 mmol) in acetone (2 mL), was added K_2CO_3 (109 mg, 0.791 mmol) and propargyl bromide (56 mg, 0.47 mmol) at rt. The reaction mixture was heated to 40 °C for 8 h. It was then cooled to rt, filtered and concentrated to dryness. Purification by chromatography on silica gel (eluting with EtOAc in cyclohexane) gave compound (2.2 g, 60% yield) as a yellow solid. ^{1}H NMR (300 MHz, $CDCl_3$) δ 9.14 (s, 1H), 8.37 – 8.21 (m, 1H), 7.68 – 7.05 (m, 7H), 5.05 (d, *J* = 2.4 Hz, 2H), 2.58 (t, *J* = 2.4 Hz, 1H). ^{13}C NMR (75 MHz, DMSO) δ 162.6, 156.5, 151.2, 143.2, 142.8, 130.8, 124.2, 124.1, 122.2, 121.8, 121.1, 120.8, 117.7, 116.5, 79.2, 57.1, 56.5. HRMS (ESI): m/z calcd for $C_{17}H_{13}N_3O^{79}Br$ [M + H]$^+$: 354.02365, found 354.0240 (1 ppm).

N-(3-Bromo-5-chlorophenyl)-8-(prop-2-yn-1-yloxy)quinazolin-2-amine (5b)

Same procedure to obtain **5b**. Yield: 0.27 g (49%). ^{1}H NMR (400 MHz, DMSO-d_6) δ 10.37 (s, 1H), 9.37 (s, 1H), 8.37 (s, 1H), 8.27 (s, 1H), 7.58 (dd, *J*= 2.0 Hz, 7.2 Hz, 1H), 7.43-7.36 (m, 2H), 7.25 (t, *J*= 1.6 Hz, 1H), 5.0 (d, *J*= 2.4 Hz, 2H), 3.66 (t, *J*= 2.4 Hz, 1H). ^{13}C NMR (75 MHz, DMSO) δ 162.7, 156.1, 151.2, 143.8, 142.7, 134.6, 124.5, 123.3, 122.6, 121.9, 120.6, 119.6, 117.2, 116.0, 79.3, 57.0. HRMS (ESI): m/z calcd for $C_{17} H_{12} N_3 O\ {}^{35}Cl\ {}^{79}Br$ [M + H]$^+$: 387.98468, found 387.9847 (0 ppm).

N-(3-Bromophenyl)-8-((1-(4-methyl-2-nitrophenyl)-1H-1,2,3-triazol-4-yl)methoxy)quinazolin-2-amine (7a)

To a solution of $CuSO_4{\cdot}5H_2O$ (6.8 mg, 0.028 mmol), sodium ascorbate (16.6 mg, 0.084 mmol) and $PhCO_2H$ (3.4 mg, 0.028 mmol) in *t*-BuOH/H_2O 1:2 (v/v, 2.0 mL) was added a mixture of alkyne **5a** (100 mg, 0.28 mmol) and aromatic azide **6** (50 mg, 0.28 mmol) at room temperature. The resultant mixture was stirred continuously until the consumption of starting material (1 h).

Then CH_2Cl_2 (20 mL) was added to dissolve the crude product. The organic layer was washed with H_2O and brine, and dried over anhydrous Na_2SO_4. Removal of the solvent yielded a residue, which was purified by a short chromatography (silica gel, EtOAc/PE 1:3) to give **7a** (108 mg, 72%) as a light yellow solid. ^{1}H NMR (400 MHz, DMSO-d$_6$) δ 10.07 (s, 1H), 9.31 (s, 1H), 8.81 (s, 1H), 8.58 (t, *J* = 2.0, 2.0 Hz, 1H), 8.04 (d, *J* = 1.8 Hz, 1H), 7.85 – 7.64 (m, 4H), 7.56 (ddd, *J* = 8.0, 4.7, 1.2 Hz, 3H), 7.37 (t, *J* = 7.9, 7.9 Hz, 1H), 7.16 (t, *J* = 8.1, 8.1 Hz, 1H), 7.10 – 6.99 (m, 1H), 5.45 (s, 3H). ^{13}C NMR (101 MHz, DMSO) δ 162.6, 156.3, 152.0, 144.2, 143.9, 142.9, 142.7, 142.5, 135.1, 130.7, 127.4, 127.0, 126.4, 126.0, 124.4, 124.1, 122.1, 121.7, 120.9, 120.6, 117.7, 116.0, 62.4, 20.9. HRMS (ESI): m/z calcd for C_{24} H_{19} N_7 O_3 ^{79}Br [M + H]$^+$: 532.07327, found 532.0723 (1 ppm).

Synthesis N-(3-bromo-5-chlorophenyl)-8-((1-(4-methyl-2-nitrophenyl)-1H-1,2,3-triazol-4-yl)methoxy)quinazolin-2-amine (7b)

Yield: 0.25 g (57%). ^{1}H NMR (400 MHz, DMSO-*d*$_6$) δ 10.34 (s, 1H), 9.38 (s, 1H), 8.88 (s, 1H), 8.26 (d, *J*= 7.2 Hz, 2H), 8.08 (s, 1H), 7.79 (d, *J*= 7.2 Hz, 1H), 7.74 (d, *J*= 8.4 Hz, 1H), 7.60 (t, *J*= 6.4 Hz, 2H), 7.42 (t, *J*= 8.0 Hz, 1H), 7.14 (t, *J*= 2.0 Hz, 1H),5.45 (s, 2H), 2.52 (s, 3H). ^{13}C NMR (101 MHz, DMSO) δ 162.2, 155.6, 151.6, 143.7, 143.3, 142.1, 141.8, 134.6, 134.0, 126.8, 126.5, 125.9, 125.6, 124.3, 122.6, 121.9, 121.4, 119.9, 119.0, 116.5, 115.2, 61.9, 20.4. MS(ESI): m/z calcd for $C_{24}H_{17}{}^{79}Br^{35}ClN_7O_3$, 566.8, found: [M+H]$^+$: 568.1.

Procedure A

Methyl 3'-((8-((1-(4-methyl-2-nitrophenyl)-1H-1,2,3-triazol-4-yl)methoxy)quinazolin-2-yl)amino)-[1,1'-biphenyl]-4-carboxylate (9a)

Under argon to a Schlenk tube equipped with a magnet stirrer, *N*-(3-bromophenyl)-8-((1-(4-methyl-2-nitrophenyl)-1*H*-1,2,3-triazol-4-yl)methoxy)quinazolin-2-amine (200 mg, 0.375 mmol; in 0.5–0.7 mL of dioxane), a solution of P(*t*-Bu)$_3$ (2.2 mg, 0.011 mmol in 3.0 mL of dioxane), $Pd_2(dba)_3$ (5.0 mg, 0.005 mmol), boronic acid (80 mg, 0.45 mmol), Cs_2CO_3 (140 mg, 0.45 mmol) are successively added and stirred for 12 h at 80 °C. The reaction mixture was then concentrated and purified by flash chromatography using EtOAc and CH_2Cl_2 to afford **9a** as a yellow compound (130 mg, 59% yield).^{1}H NMR (300 MHz, DMSO-d$_6$) δ 10.04 (s, 1H), 9.32 (s, 1H), 8.72 (s, 1H), 8.58 (t, *J* = 2.0 Hz, 1H), 8.11 – 7.98 (m, 4H), 7.90 – 7.80 (m, 2H), 7.74 (ddd, *J* = 8.1, 1.9, 0.8 Hz, 1H), 7.63 (d, *J* = 8.1 Hz, 1H), 7.56 (dq, *J* = 8.4, 1.2 Hz, 2H),

7.44 – 7.33 (m, 1H), 7.38 – 7.27 (m, 2H), 5.53 (s, 2H), 3.86 (s, 3H). ^{13}C NMR (75 MHz, DMSO-6_d) δ 166.5, 162.5, 156.8, 151.9, 145.4, 144.2, 144.0, 143.4, 142.4, 141.8, 139.4, 135.1, 130.3, 129.7, 128.9, 127.6, 127.2, 127.1, 126.3, 126.0, 123.9, 121.8, 120.6, 120.4, 119.2, 117.5, 116.4, 62.6, 52.5, 20.9. HRMS (ESI): m/z calcd for $C_{32}H_{26}$ N_7O_5 $[M + H]^+$: 588.1989, found 588.1986 (1 ppm).

Procedure B

3'-((8-((1-(4-Methyl-2-nitrophenyl)-1H-1,2,3-triazol-4-yl)methoxy)quinazolin-2-yl)amino)-[1,1'-biphenyl]-4-carboxylic acid (10a)

To a suspension of methyl 3'-((8-((1-(4-methyl-2-nitrophenyl)-1*H*-1,2,3-triazol-4-yl)methoxy) quinazolin-2-yl)amino)-[1,1'-biphenyl]-4-carboxylate (**9a**, 30 mg, 0.051 mol) in 3 mL of a THF/1,4-dioxane/water 4:4:2 mixture cooled to 0 °C was added LiOH (10 mg, 0.25 mmol, 5.0 equiv). Stirring was continued at room temperature until the starting material disappeared by TLC analysis (100% EtOAc). The reaction mixture was concentrated, diluted with water and acidified with 2 N aqueous HCl (pH 2). Then the solid formed was filtered, washed with CH_2Cl_2, and dried to obtain carboxylic acid **10a** as a yellow solid (20 mg, 68%). ^1H NMR (300 MHz, DMSO-d_6) δ 10.02 (s, 1H), 9.31 (s, 1H), 8.73 (s, 1H), 8.54 (s, 1H), 8.05 (td, *J* = 8.0, 5.9 Hz, 4H), 7.82 (d, *J* = 8.1 Hz, 2H), 7.78 – 7.60 (m, 2H), 7.55 (d, *J* = 7.9 Hz, 2H), 7.42 – 7.24 (m, 3H), 5.53 (s, 2H).). ^{13}C NMR (75 MHz, DMSO) δ 167.8, 162.5, 156.8, 151.8, 144.8, 144.2, 144.0, 143.4, 142.5, 141.8, 139.7, 135.1, 130.7, 130.4, 129.6, 127.7, 127.1, 127.0, 126.4, 126.0, 123.9, 121.7, 120.6, 120.4, 119.0, 117.5, 116.4, 62.5, 20.9. HRMS (ESI): m/z calcd for $C_{31}H_{24}N_7O_5$ $[M + H]^+$: 574.18334, found 574.1837 (1 ppm).

Methyl 3'-((8-((1-(4-methyl-2-nitrophenyl)-1H-1,2,3-triazol-4-yl)methoxy)quinazolin-2-yl)amino)-[1,1'-biphenyl]-4-carboxylate (12a)

Procedure A to afford methyl 3'-((8-((1-(4-methyl-2-nitrophenyl)-1*H*-1,2,3-triazol-4-yl)methoxy)quinazolin-2-yl)amino)-[1,1'-biphenyl]-4-carboxylate (**12a**) as a yellow compound (130 mg, 0.222 mmol, 60% yield). ^1H NMR (300 MHz, $CDCl_3$) δ 9.13 (s, 1H), 8.33 (s, 1H), 8.22 (s, 1H), 8.21 (s, 1H), 8.00 (d, *J* = 7.8 Hz, 1H), 7.92 (s, 1H), 7.85 (d, *J* = 8.6 Hz, 3H), 7.69 (s, 1H), 7.50 (d, *J* = 8.2 Hz, 2H), 7.41 (t, *J* = 7.6 Hz, 3H), 7.30 (d, *J* = 11.2 Hz, 4H), 5.62 (s, 2H), 3.95 (s, 3H), 2.53 (s, 3H). ^{13}C NMR (75 MHz, $CDCl_3$) δ 167.0 161.9, 156.5, 151.9, 144.8, 144.0, 142.0, 141.3, 140.7, 140.3, 134.2, 131.5, 130.7, 129.4, 128.8, 128.5, 128.2, 127.7, 125.8,

124.7, 123.8, 121.9, 121.2, 120.4, 118.5, 117.8, 116.5, 63.7, 52.2, 21.1. HRMS (ESI): m/z calcd for $C_{32}H_{26}$ N_7O_5 [M + H]$^+$: 588.1989, found 588.1986 (1 ppm).

3'-((8-((1-(4-Methyl-2-nitrophenyl)-1H-1,2,3-triazol-4-yl)methoxy)quinazolin-2-yl)amino)-[1,1'-biphenyl]-3-carboxylic acid (13a)

Procedure B to afford **13a** as a yellow solid (20 mg, 0.0348 mmol, 68%). ^{1}H NMR (300 MHz, DMSO) δ 10.08 (s, 1H), 9.33 (s, 1H), 8.75 (s, 1H), 8.35 (s, 1H), 8.22 (s, 1H), 8.17 (d, *J* = 8.1 Hz, 1H), 8.06 (s, 1H), 7.93 (t, *J* = 8.6 Hz, 2H), 7.75 (d, *J* = 8.2 Hz, 1H), 7.70 – 7.58 (m, 1H), 7.56 (d, *J* = 8.2 Hz, 2H), 7.35 (q, *J* = 7.4 Hz, 2H), 7.27 (d, *J* = 7.7 Hz, 1H), 5.52 (s, 2H); methyl group hidden by DSMO residual peaks. ^{13}C NMR (75 MHz, DMSO) δ 167.7, 156.7, 151.6, 144.2, 144.1, 142.9, 142.4, 141.6, 141.1, 139.9, 135.2, 131.9, 131.4, 129.8, 129.8, 128.7, 127.7, 127.7, 127.7, 127.1, 126.4, 126.1, 124.1, 120.8, 120.4, 118.8, 117.5, 116.8, 62.8, 20.9, 18.9. HRMS (ESI): m/z calcd C_{31} $H_{24}N_7O_5$ [M + H]$^+$: 574.18334, found 574.1837 (1 ppm).

Methyl-3'-chloro-5'-((8-((1-(4-methyl-2-nitrophenyl)-1H-1,2,3-triazol-4-yl) methoxy)quinazolin-2-yl)amino)-[1,1'-biphenyl]-3-carboxylate (9b)

Procedure A to afford **9b** a cream colour (very light yellow) compound (40 mg, 0,064 mmol, 60% yield). ^{1}H NMR (300 MHz, DMSO) δ 10.23 (s, 1H), 9.35 (s, 1H), 8.77 (s, 1H), 8.44 (t, *J* = 1.9 Hz, 1H), 8.29 (t, *J* = 1.7 Hz, 1H), 8.04 (dd, *J* = 8.7, 2.0 Hz, 3H), 7.84 (d, *J* = 8.6 Hz, 1H), 7.75 (ddd, *J* = 8.2, 1.9, 0.8 Hz, 1H), 7.65 (d, *J* = 8.1 Hz, 1H), 7.58 (ddd, *J* = 8.2, 4.5, 1.2 Hz, 2H), 7.32 (t, *J* = 1.7 Hz, 1H), 5.74 (s, 0H), 5.51 (s, 2H), 3.86 (s, 3H). ^{13}C NMR (75 MHz, DMSO) δ 166.4, 162.7, 156.4, 152.0, 144.2, 143.9, 143.9, 143.1, 142.9, 142.4, 141.1, 135.1, 134.4, 130.3, 129.4, 127.4, 127.4, 127.0, 126.3, 126.0, 124.5, 121.9, 120.5, 119.6, 117.9, 116.0, 115.8, 62.5, 52.6, 20.9. HRMS (ESI): m/z calcd for $C_{32}H_{25}N_7O_5$ ^{35}Cl [M + H]$^+$: 622.16002, found 622.1598 (0 ppm).

3'-Chloro-5'-((8-((1-(4-methyl-2-nitrophenyl)-1H-1,2,3-triazol-4-yl)methoxy)quinazolin-2-yl)amino)-[1,1'-biphenyl]-4-carboxylic acid (10b)

Procedure B to obtain carboxylic acid **10b** as a yellow solid (10 mg, 60% yield). ^{1}H NMR (400 MHz, DMSO) δ 10.25 (s, 1H), 9.35 (s, 1H), 8.80 (s, 1H), 8.47 (s, 1H), 8.25 (s, 1H), 8.10 – 7.96 (m, 3H), 7.81 (d, *J* = 8.1 Hz, 2H), 7.75 (dd, *J* = 8.2, 1.7 Hz, 1H), 7.66 (d, *J* = 8.1 Hz, 1H), 7.59

(dd, J = 7.9, 3.9 Hz, 2H), 7.39 (t, J = 7.8 Hz, 1H), 7.30 (s, 1H), 5.50 (s, 2H). ^{13}C NMR (101 MHz, DMSO) δ 166.5, 162.7, 156.4, 152.0, 144.2, 143.9, 143.1, 143.0, 142.4, 141.3, 139.9, 135.1, 134.5, 131.9, 130.9, 130.1, 129.1, 127.5, 127.4, 127.1, 126.2, 126.0, 124.4, 121.9, 120.5, 119.4, 117.5, 116.1, 115.7, 62.6, 52.8, 20.9. HRMS (ESI): m/z calcd for $C_{31}H_{21}N_7O_5$ ^{35}Cl [M + H]$^-$: 606.12982, found 606.1299 (0 ppm) (1 ppm).

Methyl-3'-chloro-5'-((8-((1-(4-methyl-2-nitrophenyl)-1H-1,2,3-triazol-4-yl)methoxy)quinazolin-2-yl)amino)-[1,1'-biphenyl]-3-carboxylate (12b)

Procedure A to afford methyl 3'-chloro-5'-((8-((1-(4-methyl-2-nitrophenyl)-1*H*-1,2,3-triazol-4-yl)methoxy)quinazolin-2-yl)amino)-[1,1'-biphenyl]-3-carboxylate a cream colour (very light yellow) compound **12b** (40 mg, 60% yield). ^{1}H NMR (400 MHz, DMSO) δ 10.26 (s, 1H), 9.37 (s, 1H), 8.80 (s, 1H), 8.58 (t, J = 1.9 Hz, 1H), 8.29 – 8.04 (m, 3H), 7.97 (ddt, J = 8.9, 3.7, 1.2 Hz, 2H), 7.77 (ddd, J = 8.1, 1.9, 0.8 Hz, 1H), 7.72 – 7.48 (m, 4H), 7.44 – 7.34 (m, 1H), 7.29 (t, J = 1.8 Hz, 1H), 5.51 (s, 2H), 3.90 (s, 3H), 2.55 – 2.35 (m, 5H). ^{13}C NMR (101 MHz, DMSO) δ 166.5, 162.7, 156.4, 152.0, 144.2, 143.9, 143.1, 143.0, 142.4, 141.3, 139.9, 135.1, 134.5, 131.9, 130.9, 130.1, 129.1, 127.5, 127.4, 127.1, 126.2, 126.0, 124.4, 121.9, 120.5, 119.4, 117.5, 116.1, 115.7, 62.6, 52.8, 20.9. HRMS (ESI): m/z calcd for $C_{32}H_{25}N_7O_5{}^{35}Cl$ [M + H]$^+$: 622.16002, found 622.1598 (0 ppm).

3'-Chloro-5'-((8-((1-(4-methyl-2-nitrophenyl)-1H-1,2,3-triazol-4-yl)methoxy)quinazolin-2-yl)amino)-[1,1'-biphenyl]-3-carboxylic acid (13b)

Procedure B to obtain carboxylic acid **13b** as a yellow solid (10 mg, 70%). ^{1}H NMR (400 MHz, DMSO) δ 10.24 (s, 1H), 9.36 (s, 1H), 8.79 (s, 1H), 8.59 (d, J = 2.1 Hz, 1H), 8.21 (t, J = 1.9 Hz, 1H), 8.14 – 8.03 (m, 2H), 8.02 – 7.90 (m, 2H), 7.77 (dd, J = 8.4, 1.9 Hz, 1H), 7.69 (d, J = 8.1 Hz, 1H), 7.66 – 7.55 (m, 3H), 7.40 (t, J = 7.9 Hz, 1H), 7.27 (t, J = 1.8 Hz, 1H), 5.50 (s, 2H), 3.59 – 3.55 (m, 4H), 2.57 (s, 1H). ^{13}C NMR (101 MHz, DMSO) δ 167.8, 162.7, 156.4, 152.0, 144.2, 143.9, 143.1, 143.0, 142.4, 141.5, 139.7, 135.1, 134.5, 132.3, 131.3, 129.9, 129.3, 127.7, 127.4, 127.0, 126.2, 126.1, 124.5, 121.8, 120.5, 119.3, 117.4, 116.0, 115.7, 62.5, 20.9. m/z calcd for $C_{31}H_{21}N_7O_5{}^{35}Cl$ [M-H]$^-$: 606.12982, 606.1290 (1 ppm).

2- Kinase inhibition studies

Kinase enzymatic activities were assayed using both luminescent ADP detection assay (ADP-Glo^{TM} assay kit, Promega, Madison, WI) or radiometric kinase assay. These assays were performed using the protocols described in our previous publication [1,2].

3- Molecular modelling studies

These studies were performed using the methods described in our previous publications [1,2]. Crystal structures used for docking: CLK3: (PDB ID :2WU6) https://doi.org/10.2210/pdb2WU6/pdb, [3]) and DYRKs: (PDB ID :8T2H) https://doi.org/10.2210/pdb8T2H/pdb [4]).

References

1. Brahmaiah, D.; Kanaka Durga Bhavani, A.; Aparna, P.; Sampath Kumar, N.; Solhi, H.; Le Guevel, R.; Baratte, B.; Ruchaud, S.; Bach, S.; Singh Jadav, S.; Raji Reddy, C.; Roisnel, T.; Mosset, P.; Levoin, N.; Grée, R. *Bioorg. Med. Chem.* **2021,** *31,* 115962. doi:10.1016/j.bmc.2020.115962

2. Brahmaiah, D.; Bhavani, A. K. D.; Aparna, P.; Kumar, N. S.; Solhi, H.; Le Guevel, R.; Baratte, B.; Robert, T.; Ruchaud, S.; Bach, S.; Jadav, S. S.; Reddy, C. R.; Mosset, P.; Gouault, N.; Levoin, N.; Grée, R. *Molecules* **2022,** *27,* 6149. doi:10.3390/molecules27196149

3. Fedorov, O.; Huber, K.; Eisenreich, A.; Filippakopoulos, P.; King, O.; Bullock, A. N.; Szklarczyk, D.; Jensen, L. J.; Fabbro, D.; Trappe, J.; Rauch, U.; Bracher, F.; Knapp, S. *Chem. Biol.* **2011,** *18,* 67–76. doi:10.1016/j.chembiol.2010.11.009

4. Wilms, G.; Schofield, K.; Maddern, S.; Foley, C.; Shaw, Y.; Smith, B.; Basantes, L. E.; Schwandt, K.; Babendreyer, A.; Chavez, T.; McKee, N.; Gokhale, V.; Kallabis, S.; Meissner, F.; Rokey, S. N.; Dunckley, T.; Montfort, W. R.; Becker, W.; Hulme, C. *J. Med. Chem.* **2024,** *67,* 17259–17289. doi:10.1021/acs.jmedchem.4c01130

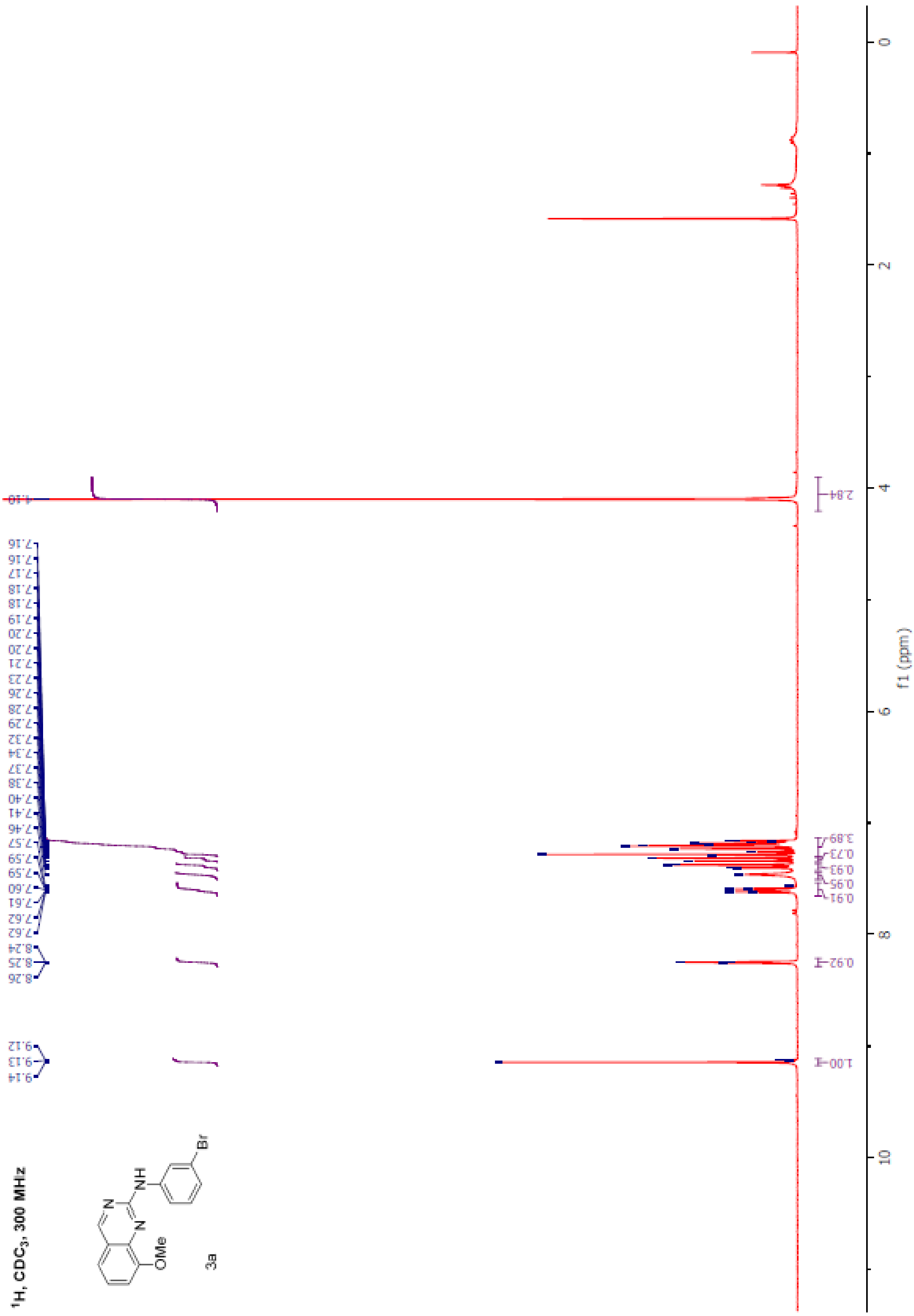

Figure S2: ^{1}H NMR spectrum of **3a**

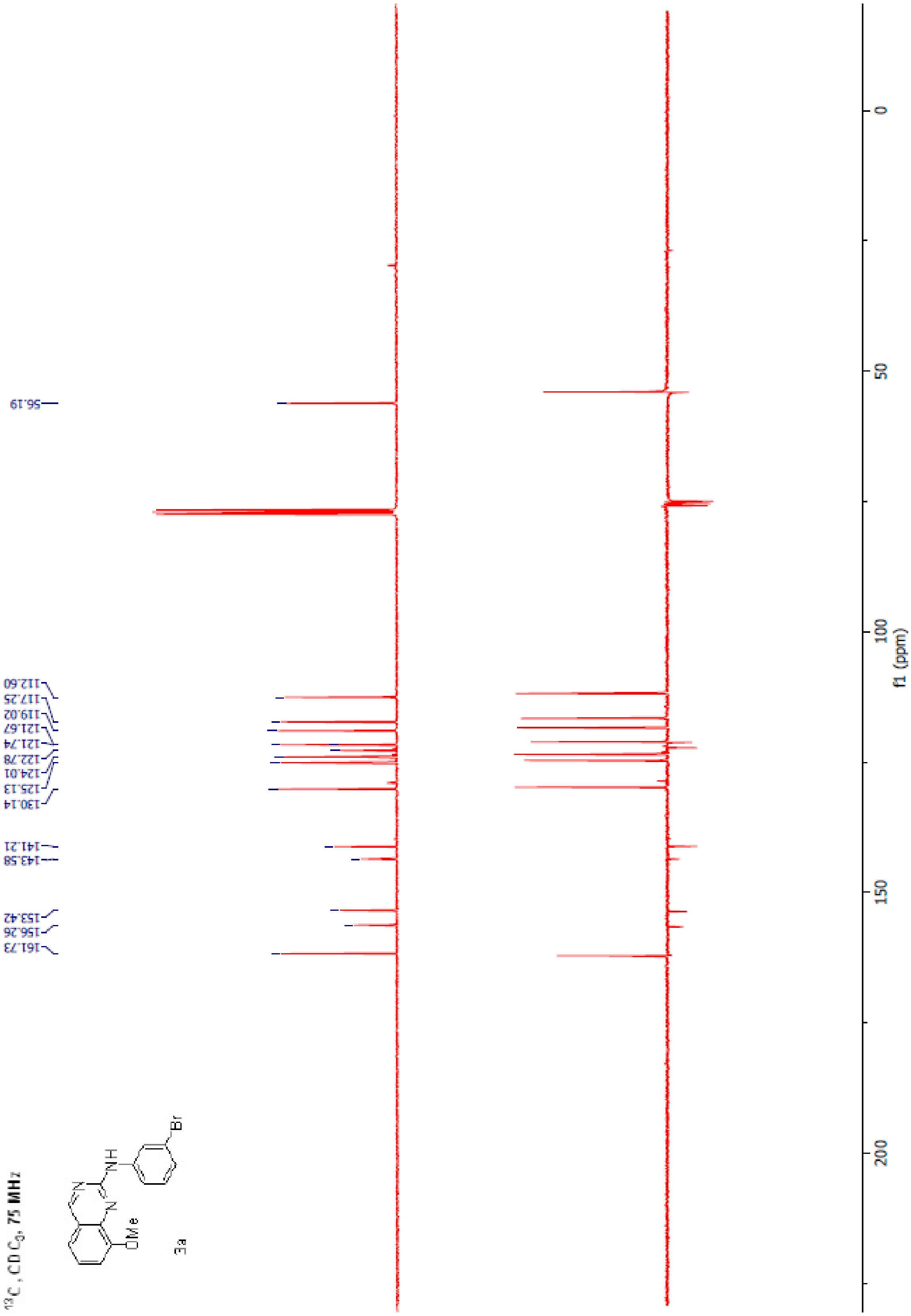

Figure S3: ^{13}C NMR spectra of **3a**

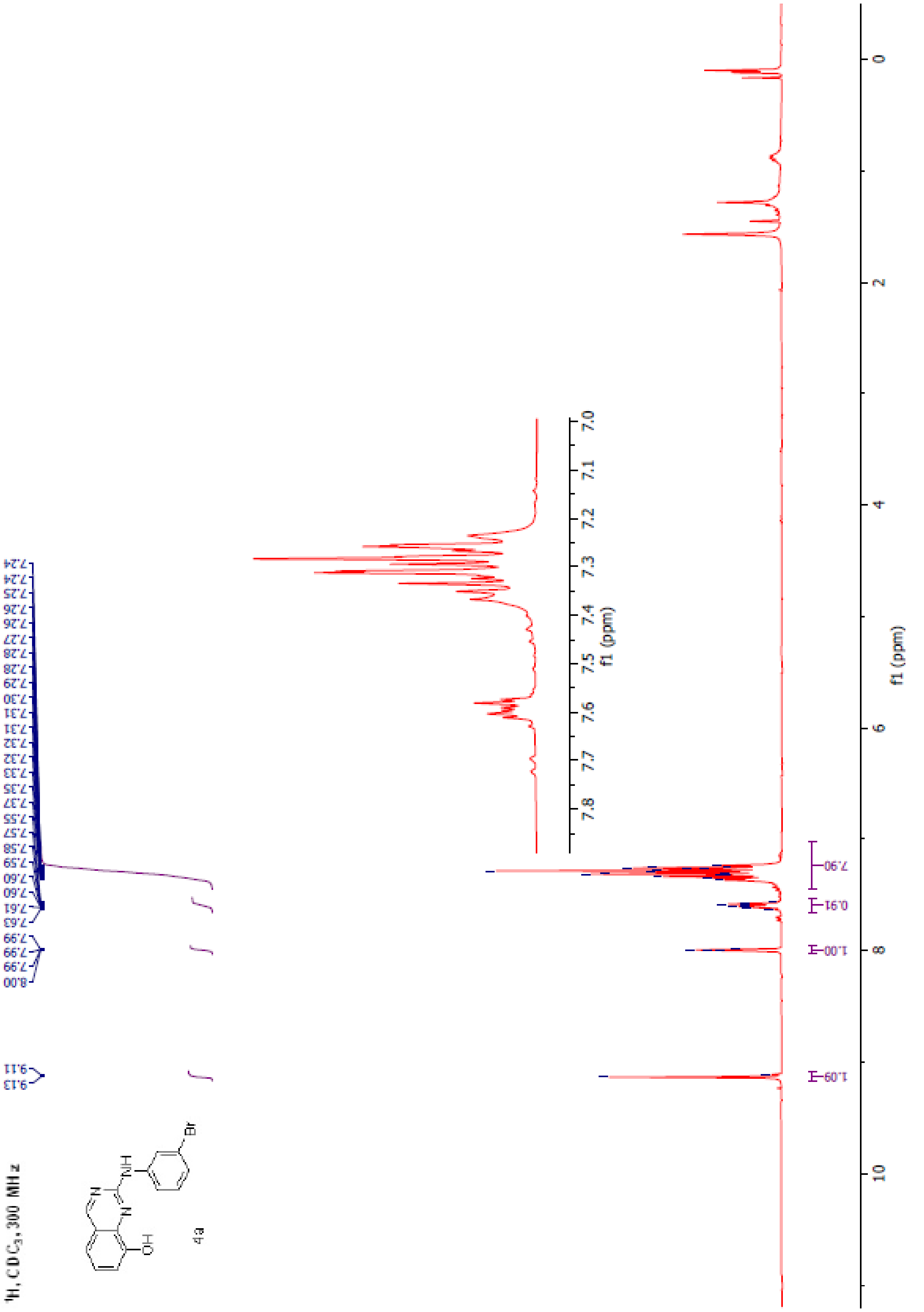

Figure S4: ^{1}H NMR spectrum of **4a**

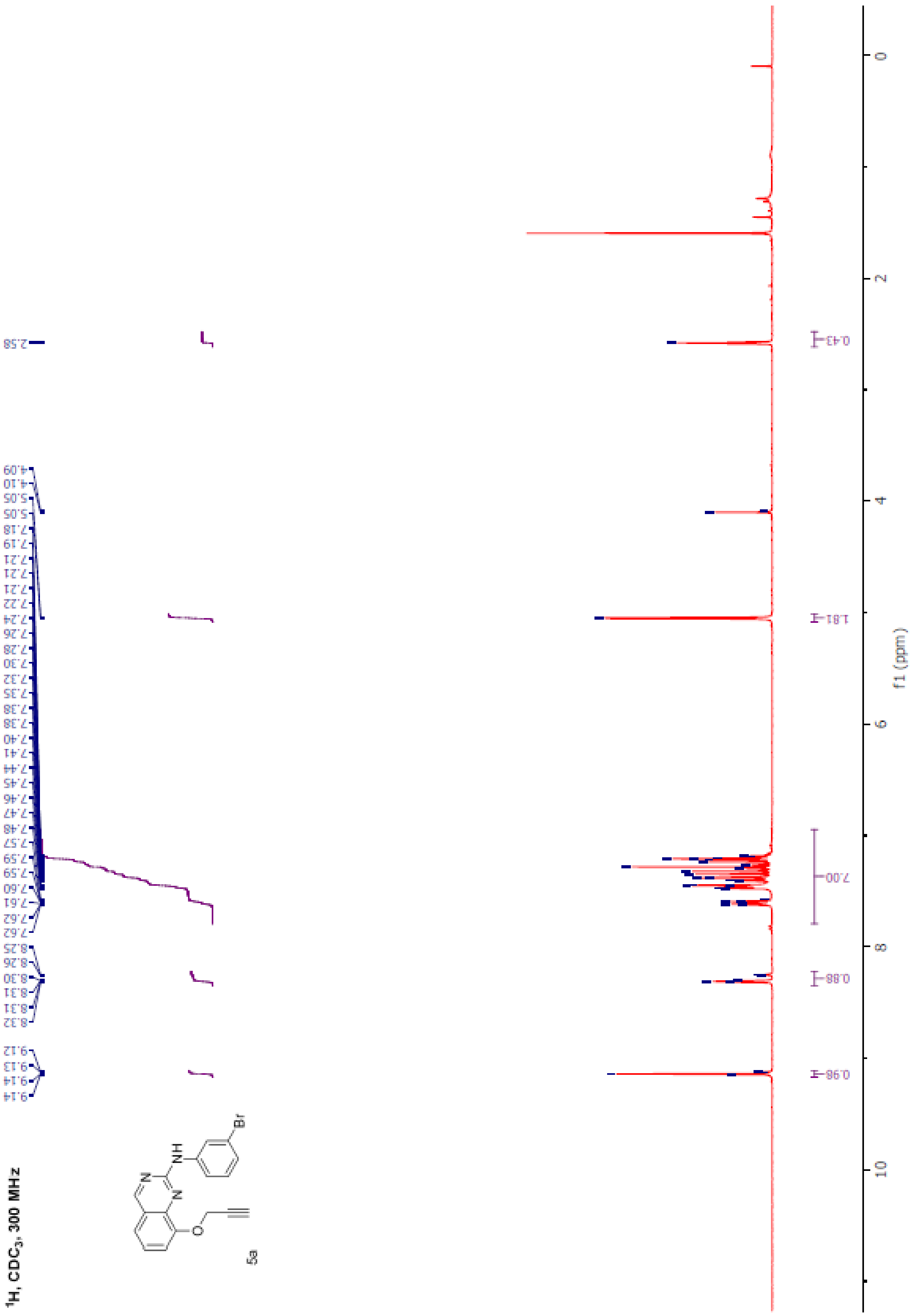

Figure S5: ^{1}H spectrum of **5a**

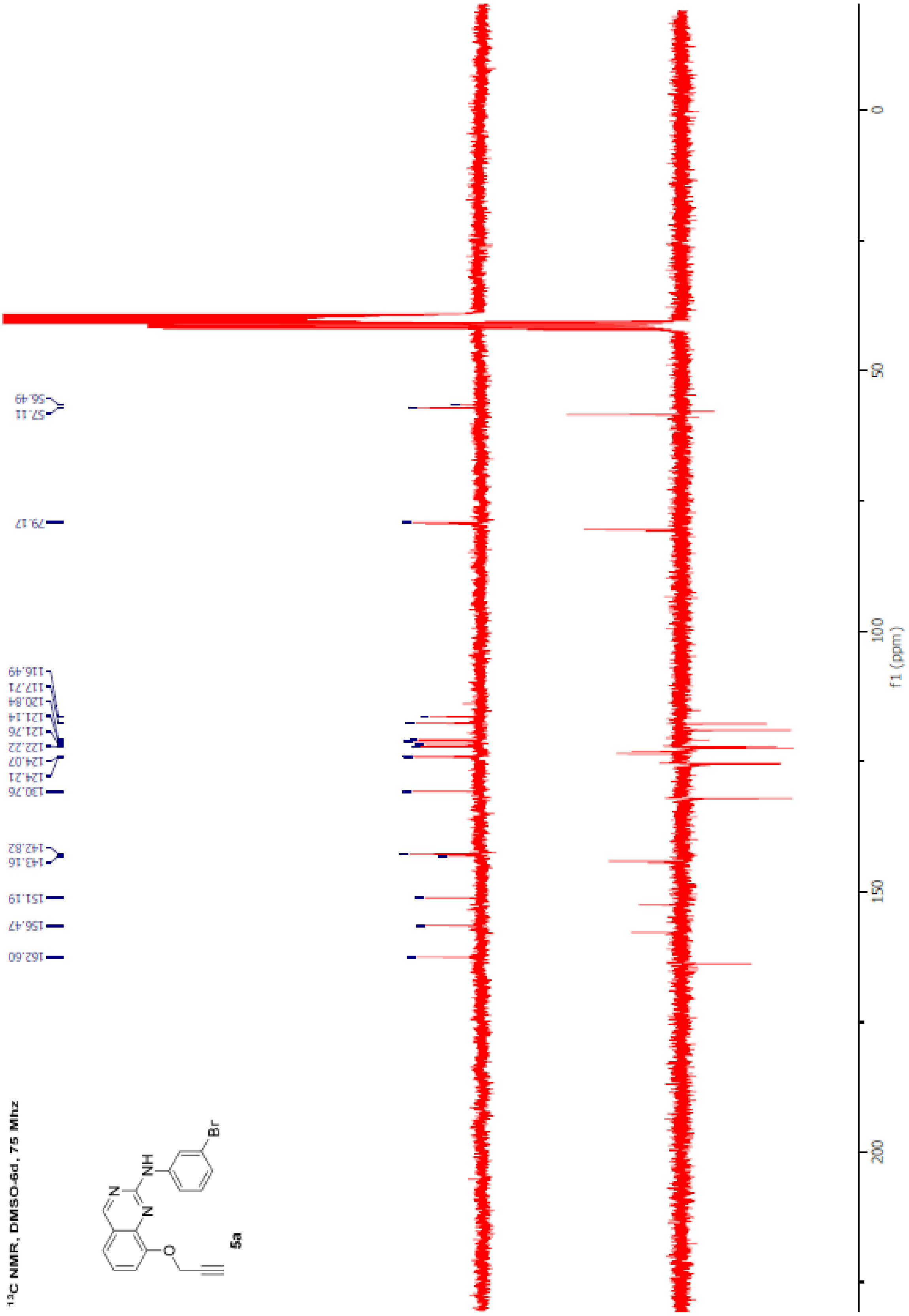

Figure S6: ^{13}C NMR spectrum of **5a**

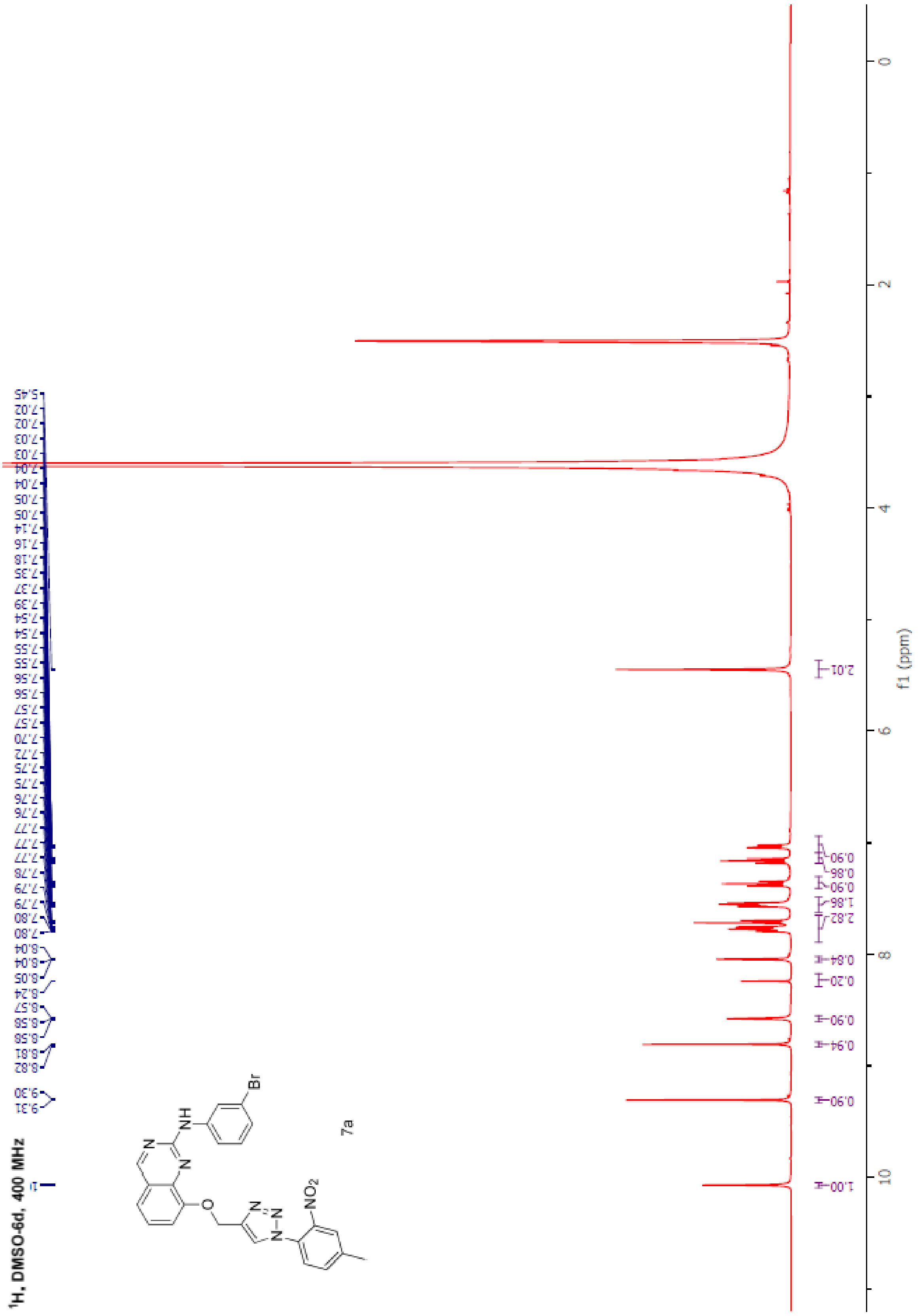

Figure S7: ¹H NMR spectrum of **7a**

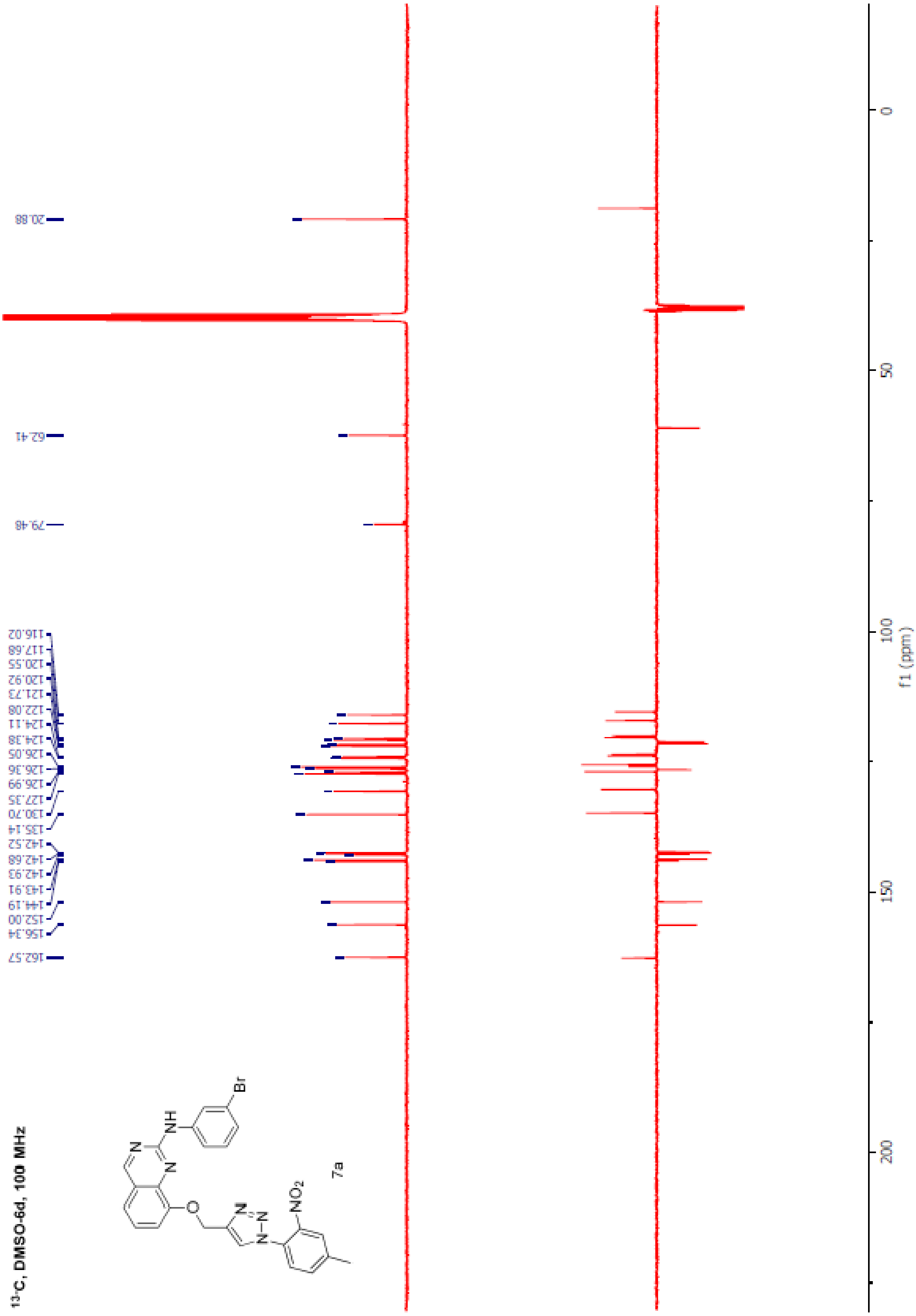

Figure S8: ^{13}C NMR spectra of **7a**

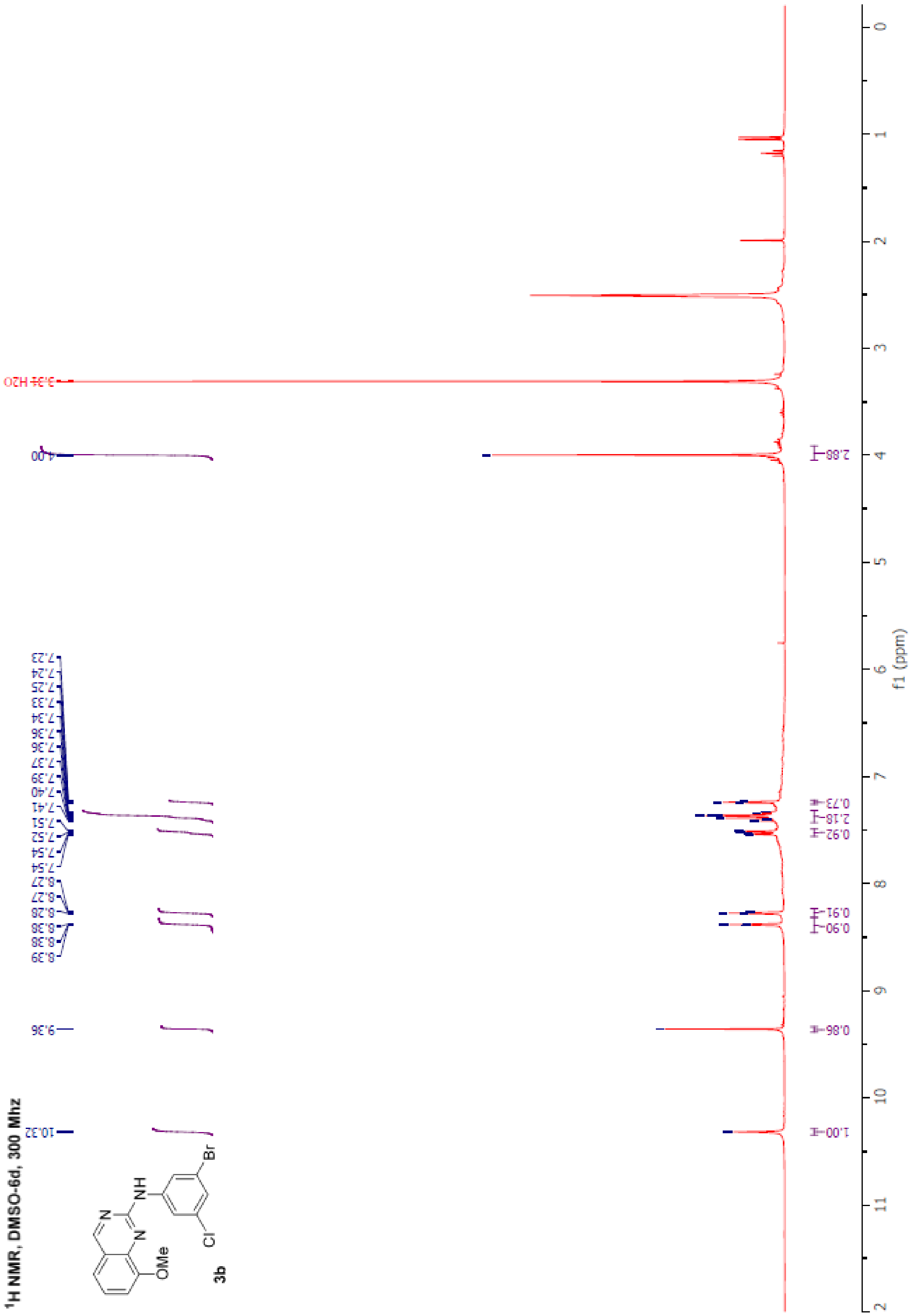

Figure S9: ^{1}H NMR spectrum of **3b**

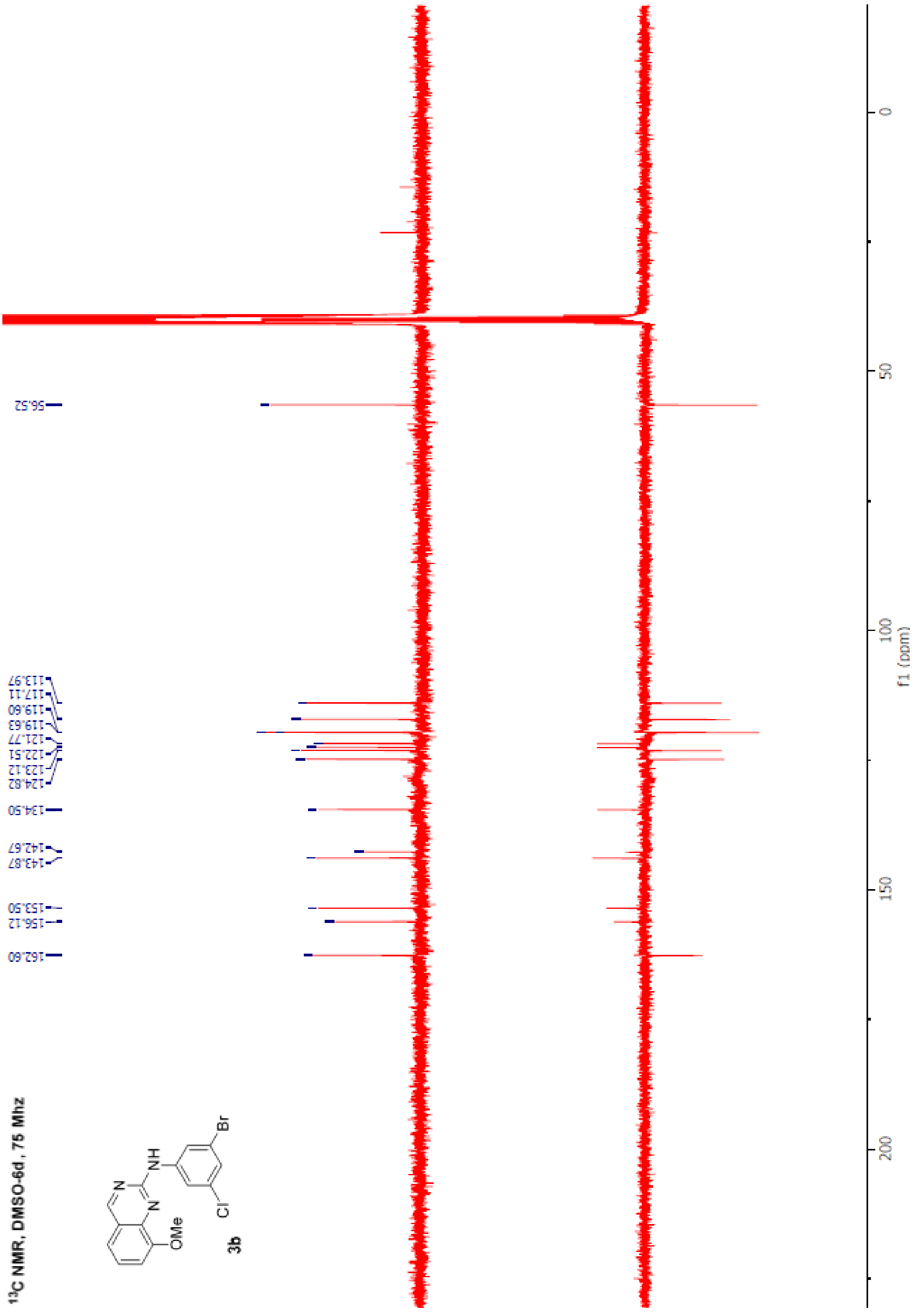

Figure S10: ^{13}C NMR spectra of **3b**

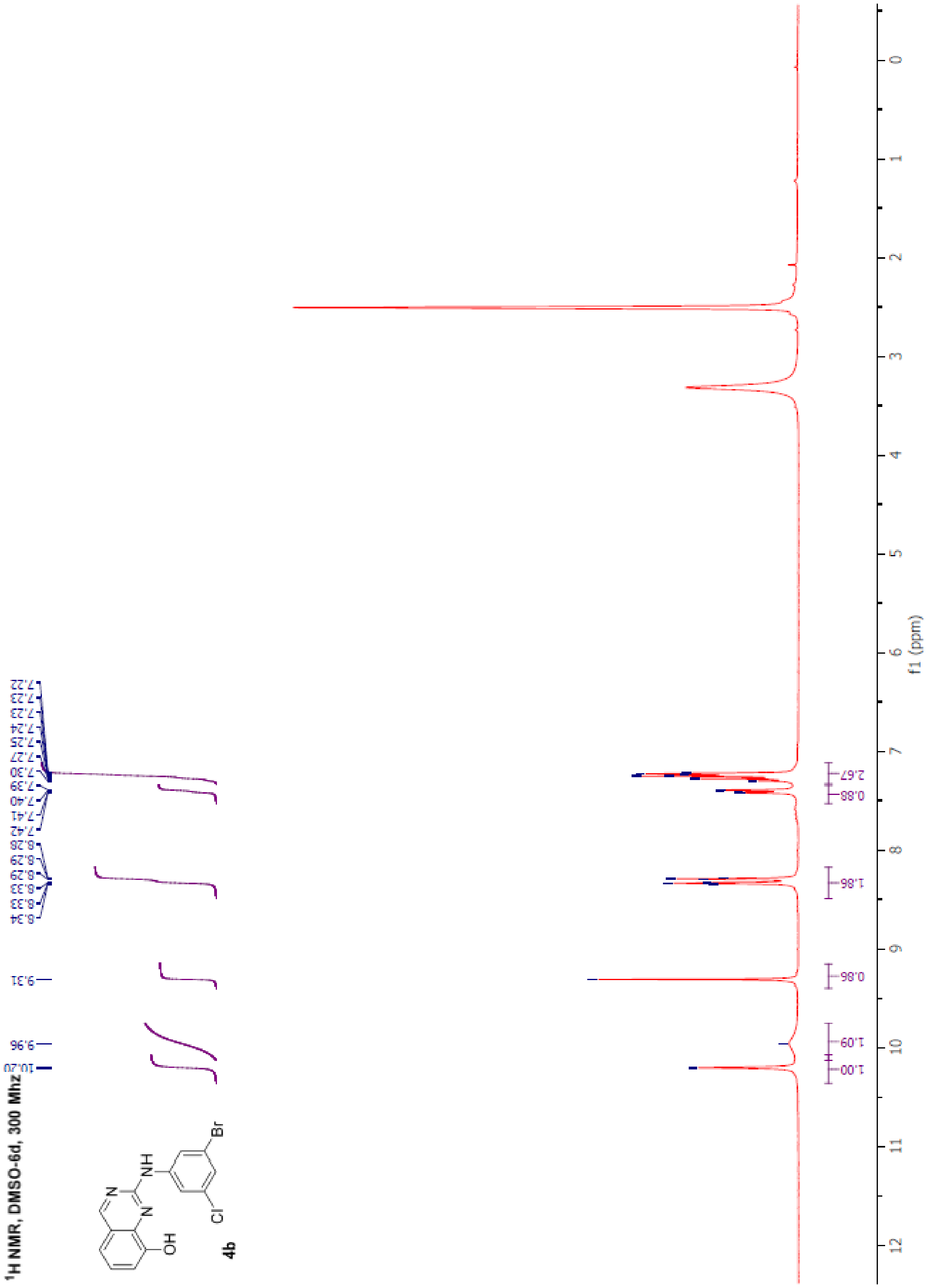

Figure S11: ^{1}H NMR spectrum of **4b**

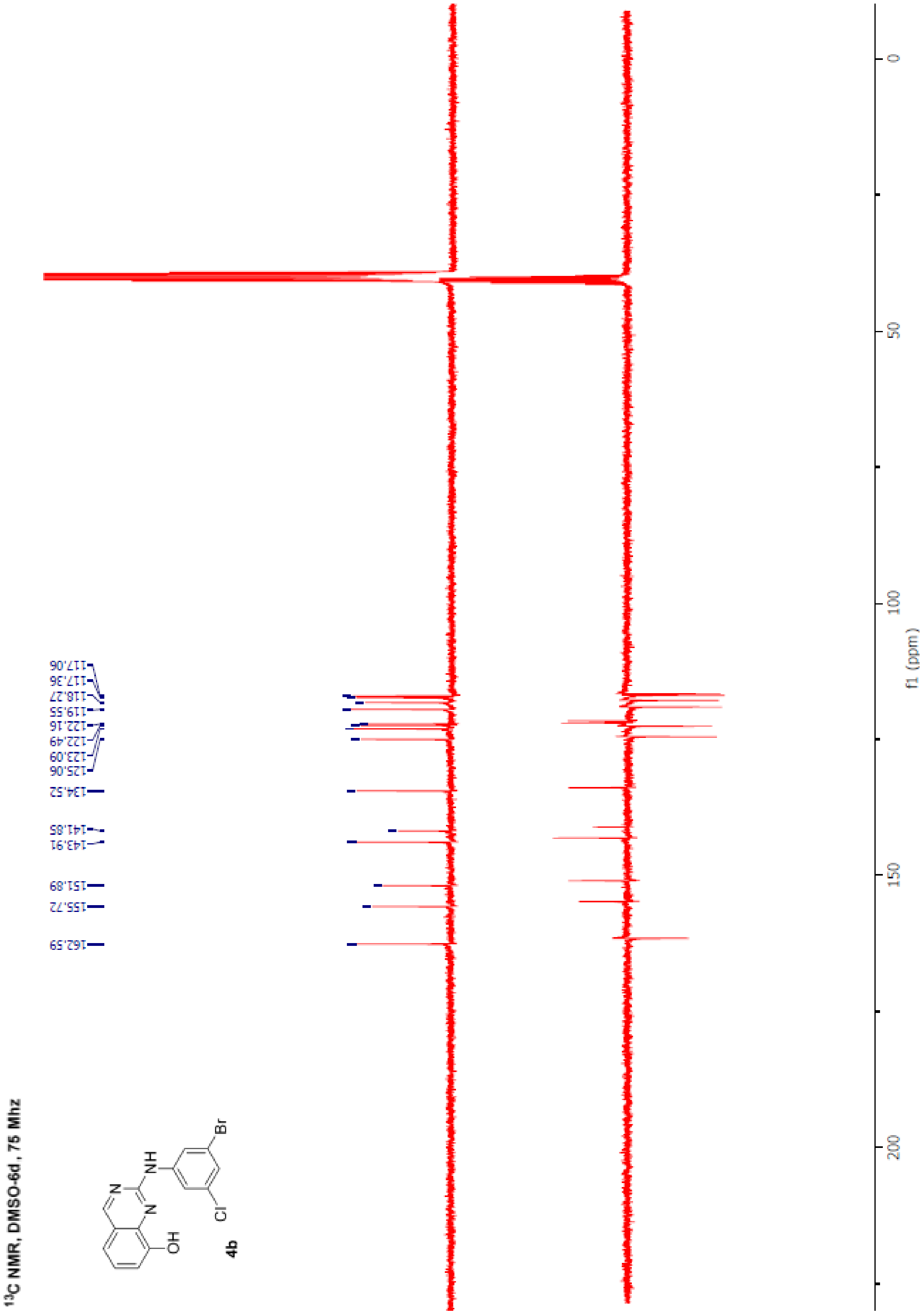

Figure S12: ^{13}C NMR spectra of **4b**

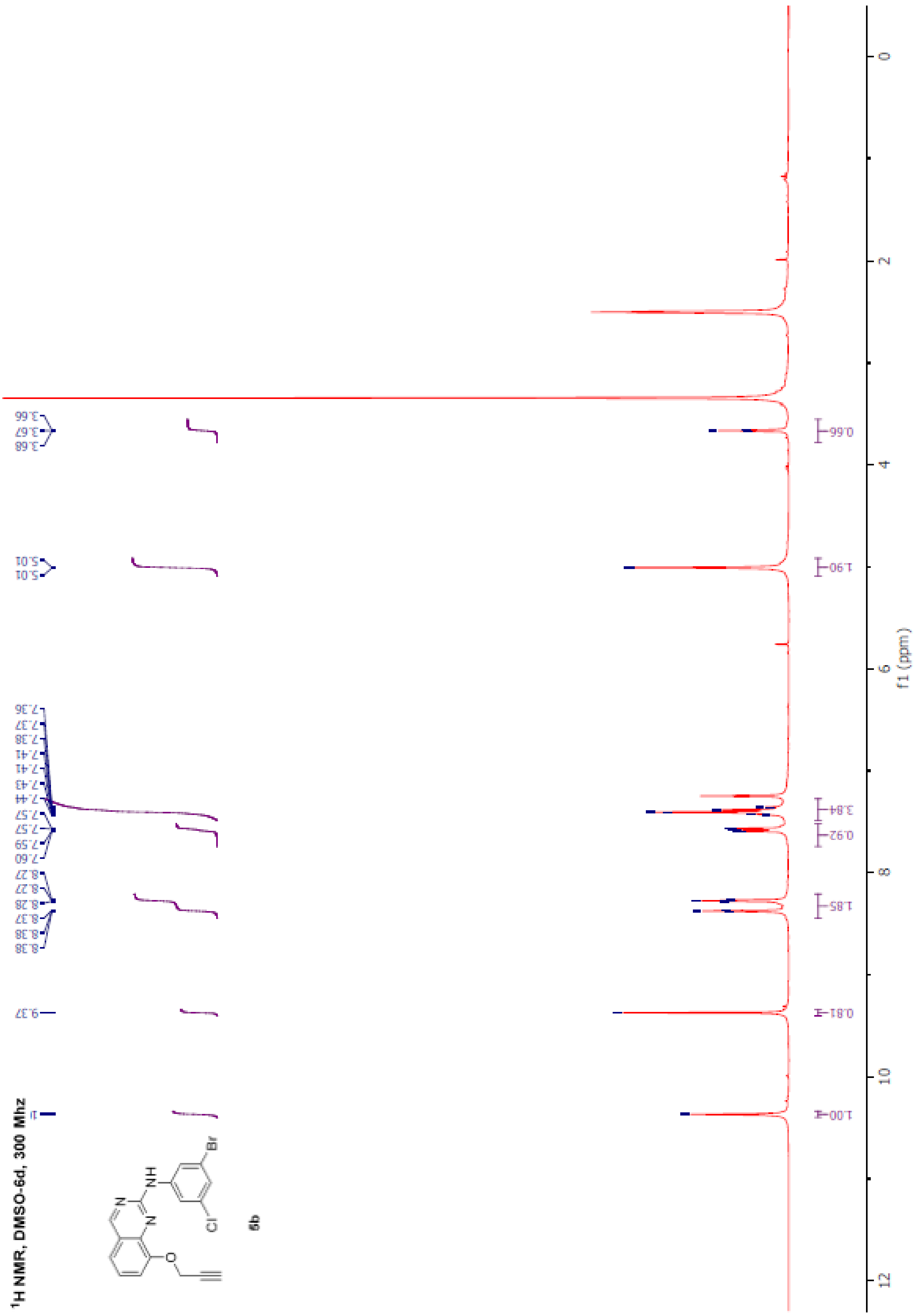

Figure S13: ^{1}H NMR spectrum of **5b**

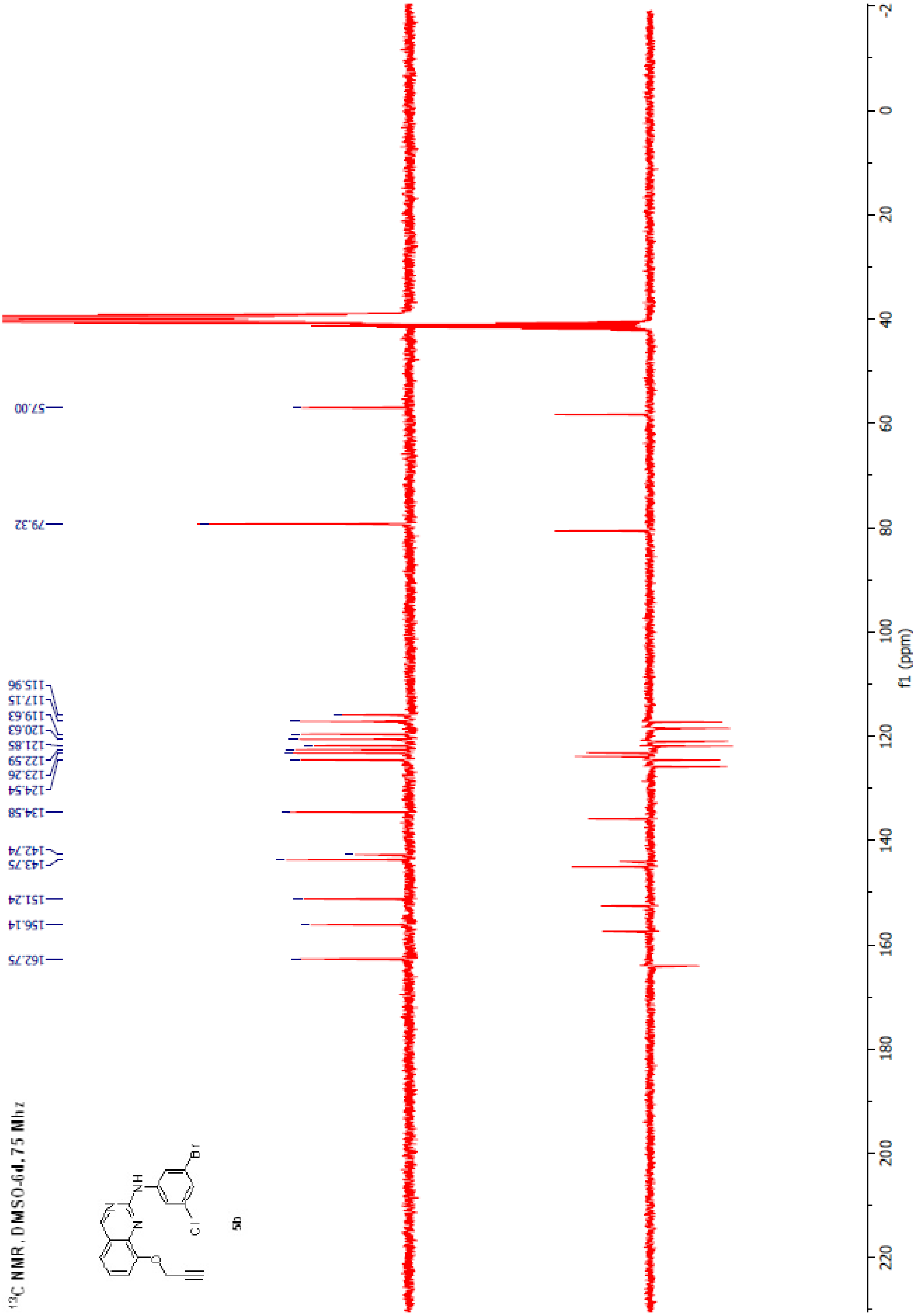

Figure S14: ^{13}C NMR spectra of **5b**

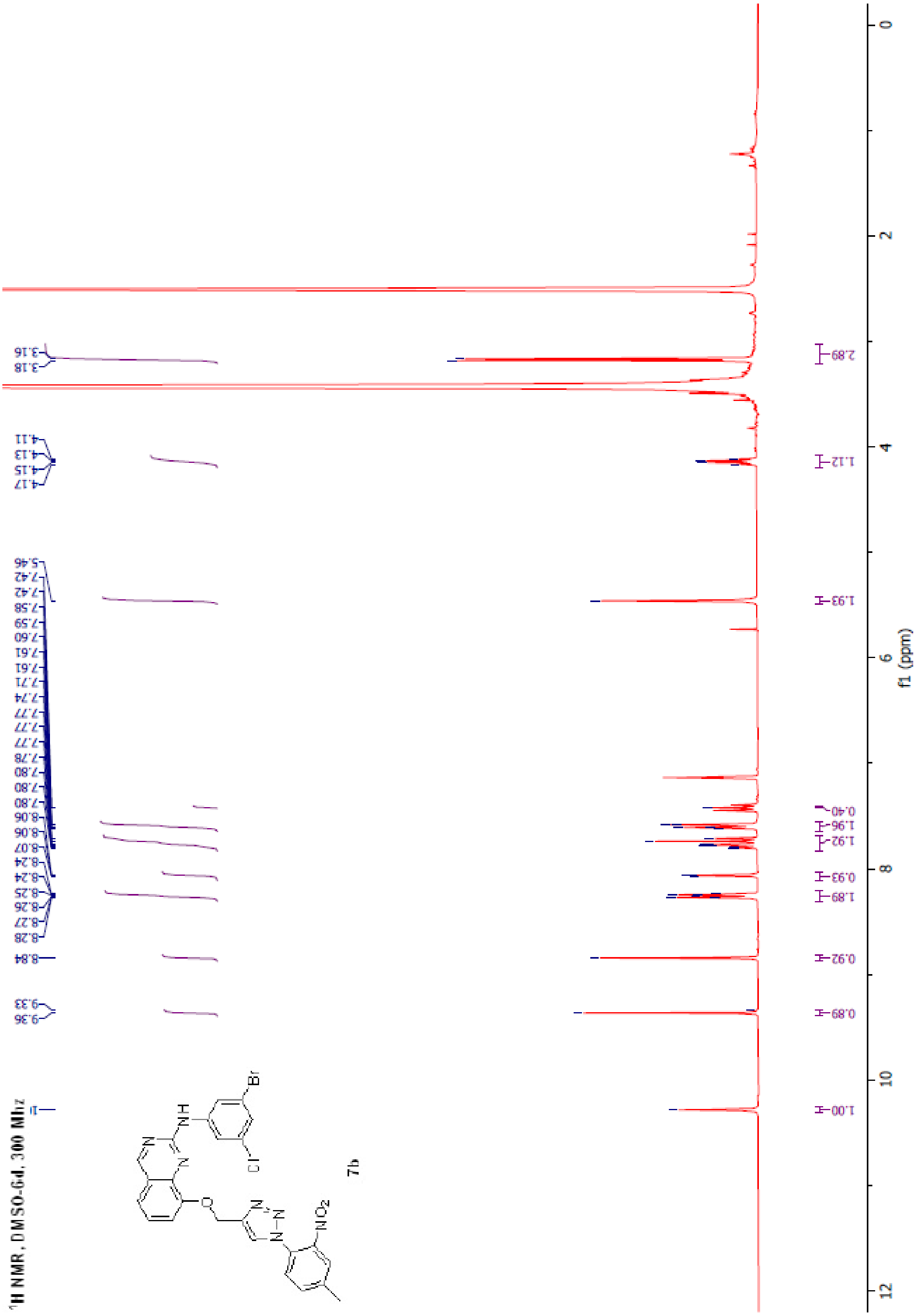

Figure S15: ^{1}H NMR spectrum of **7b**

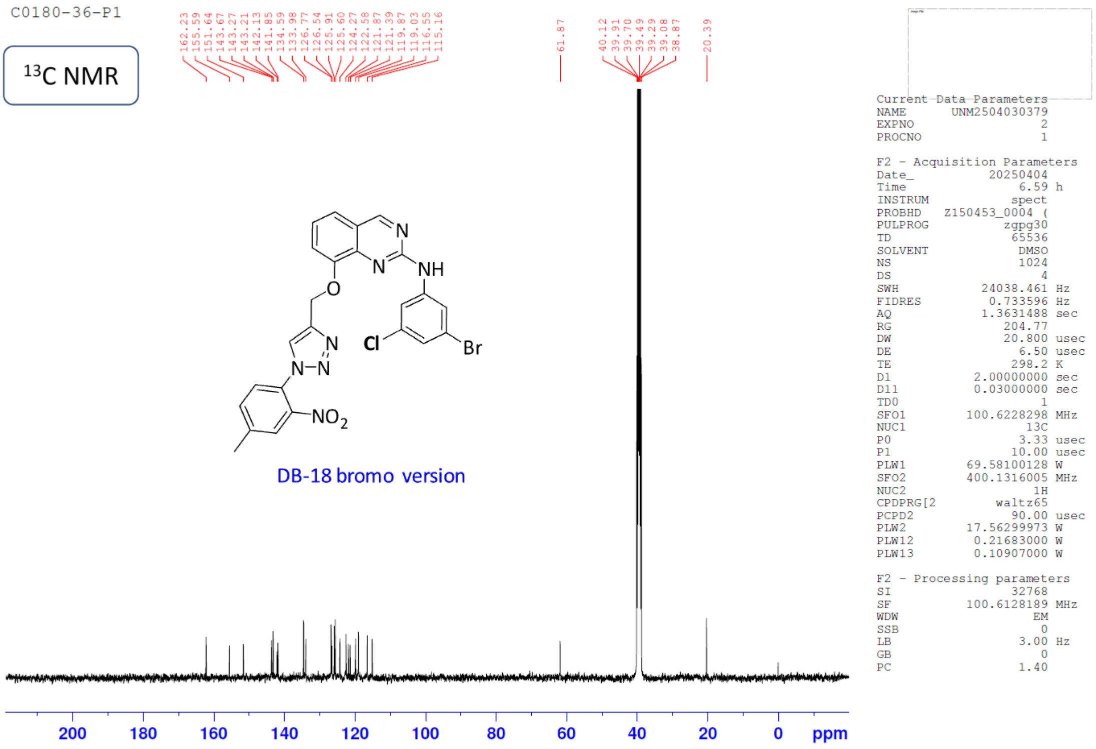

Figure S16: ^{13}C NMR spectrum of **7b**

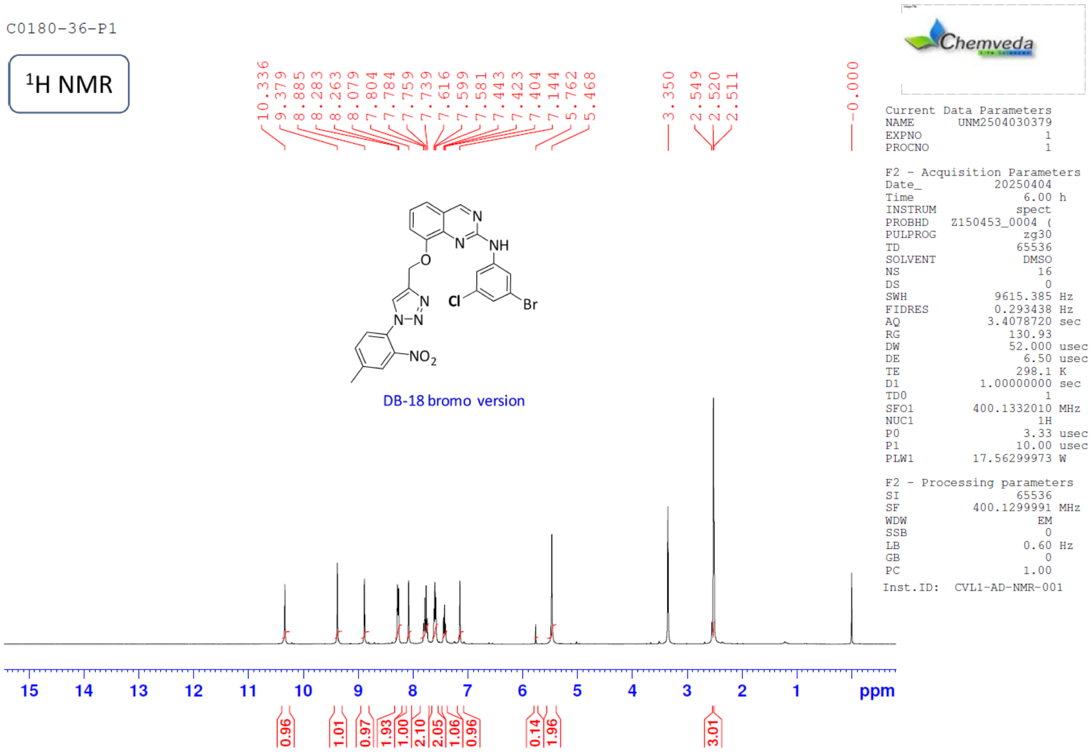

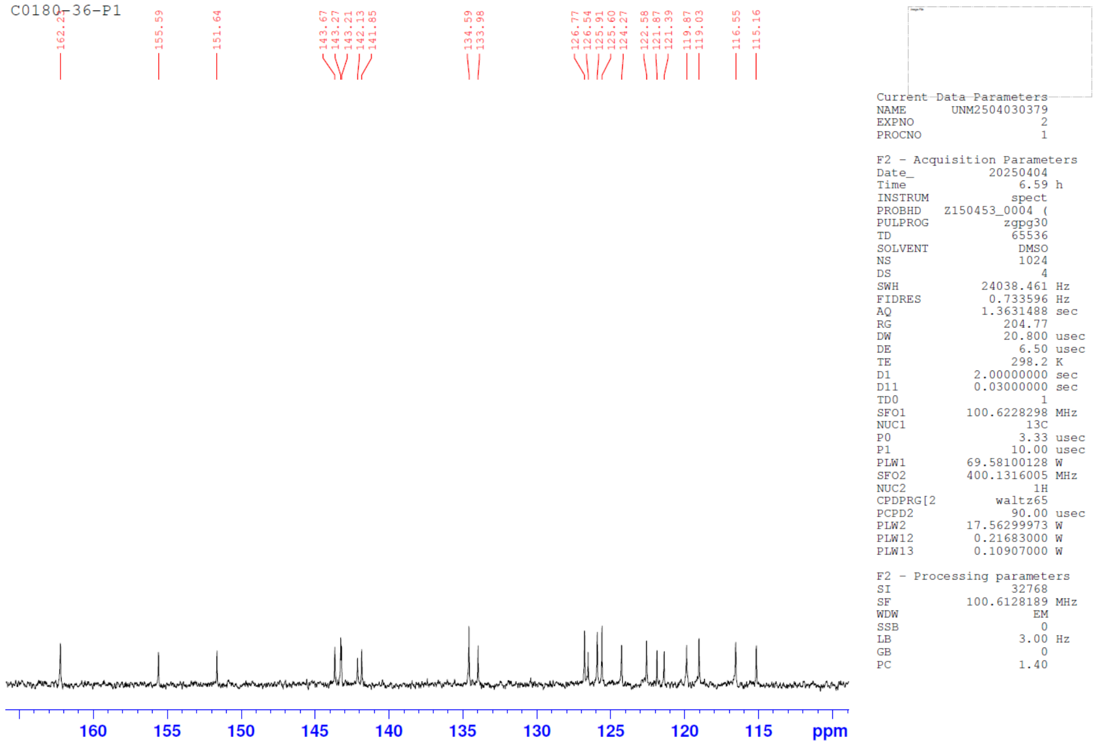

Figure S17: Extension of ^{13}C NMR spectrum of **7b**

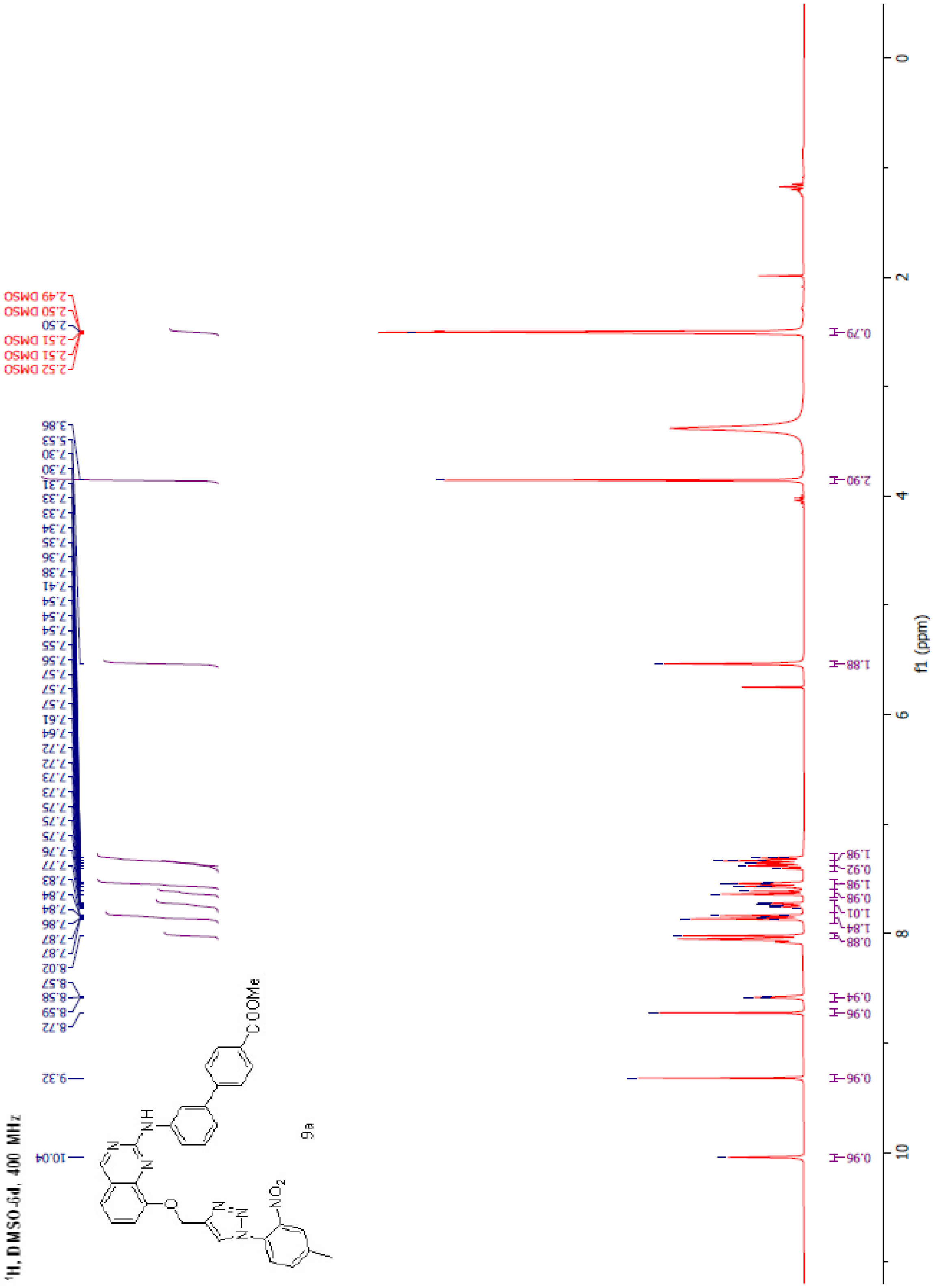

Figure S18: ^{1}H NMR spectrum of **9a**

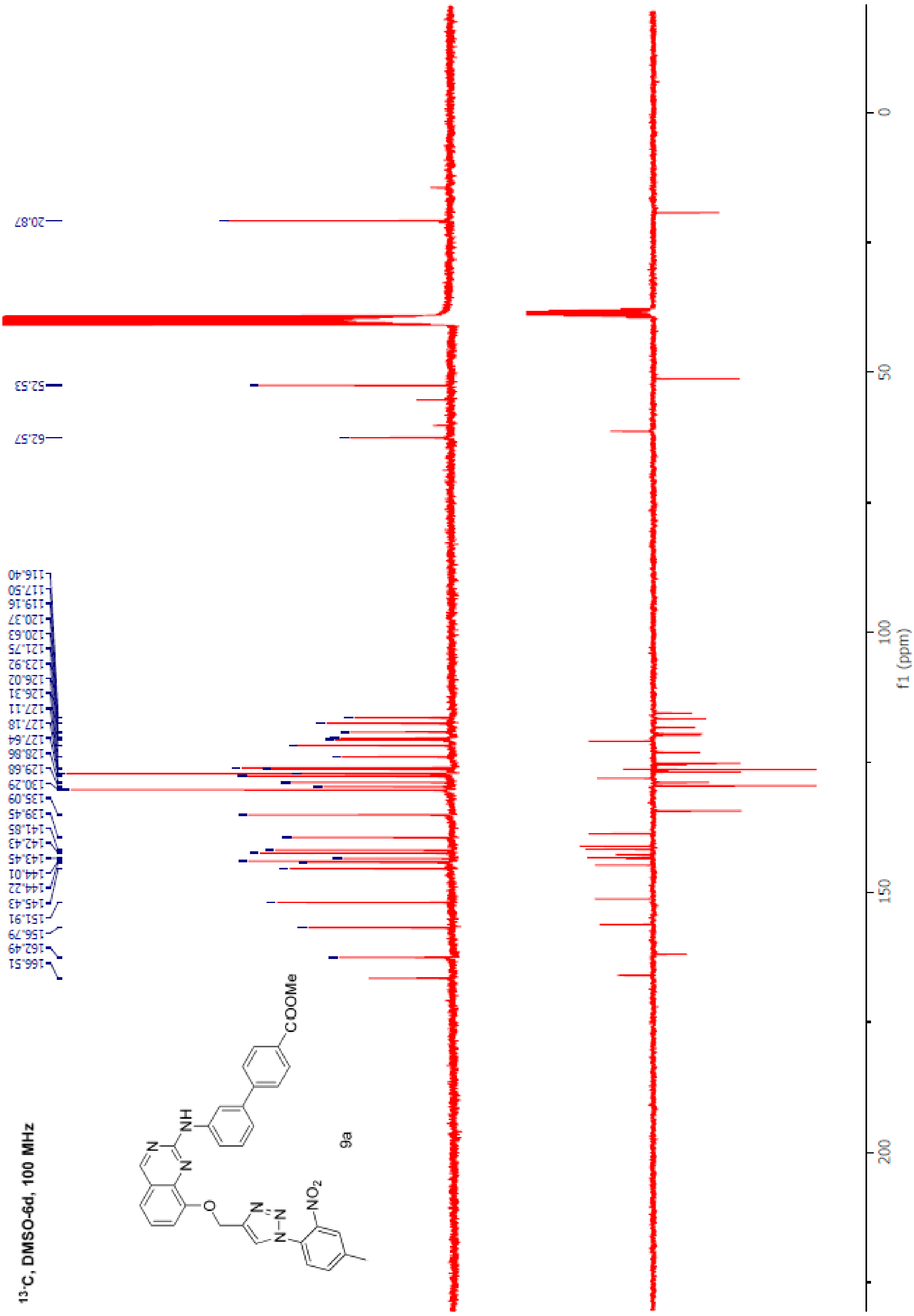

Figure S19: ^{13}C NMR spectra of **9a**

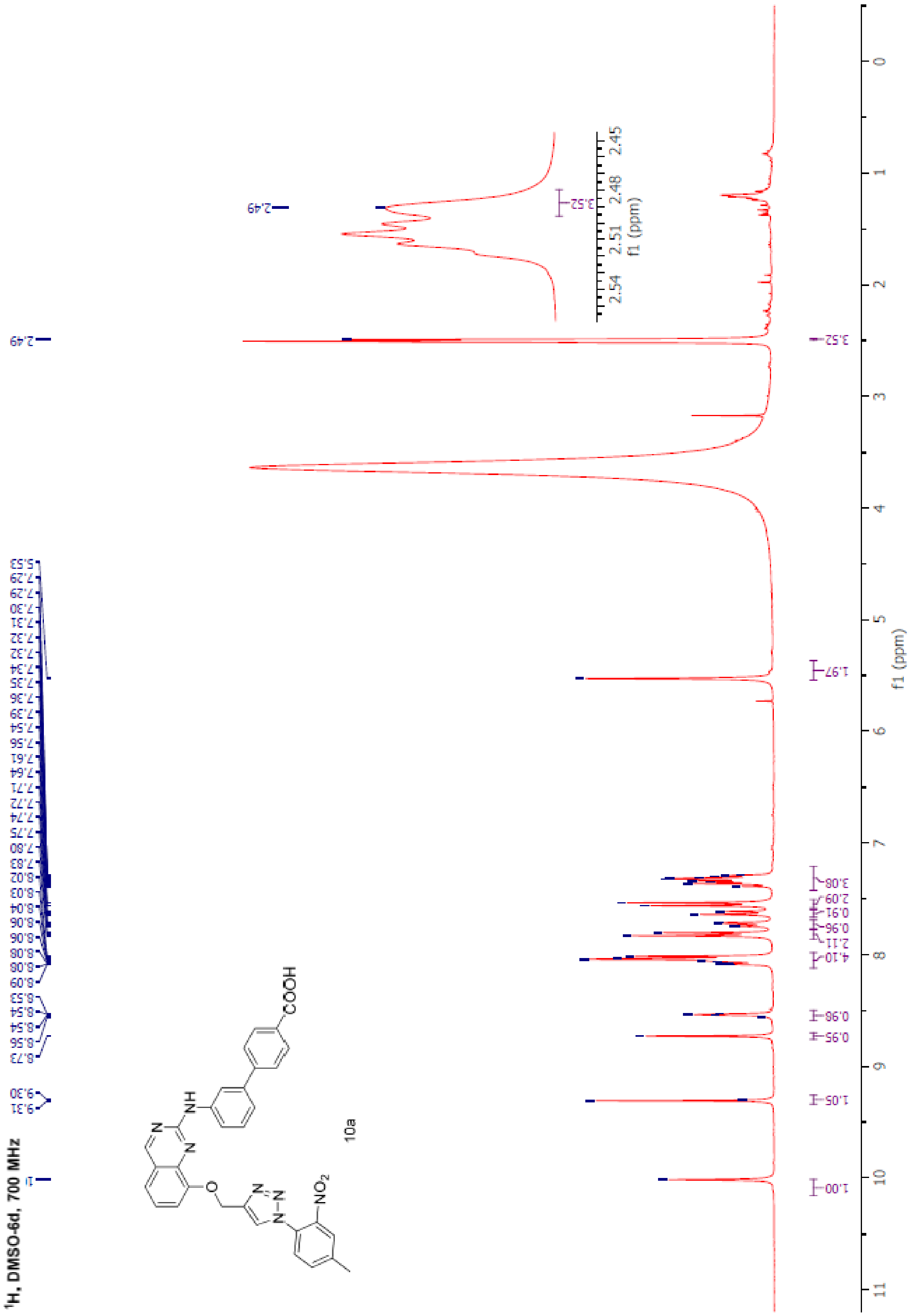

Figure S20: ^{1}H NMR spectra of **10a**

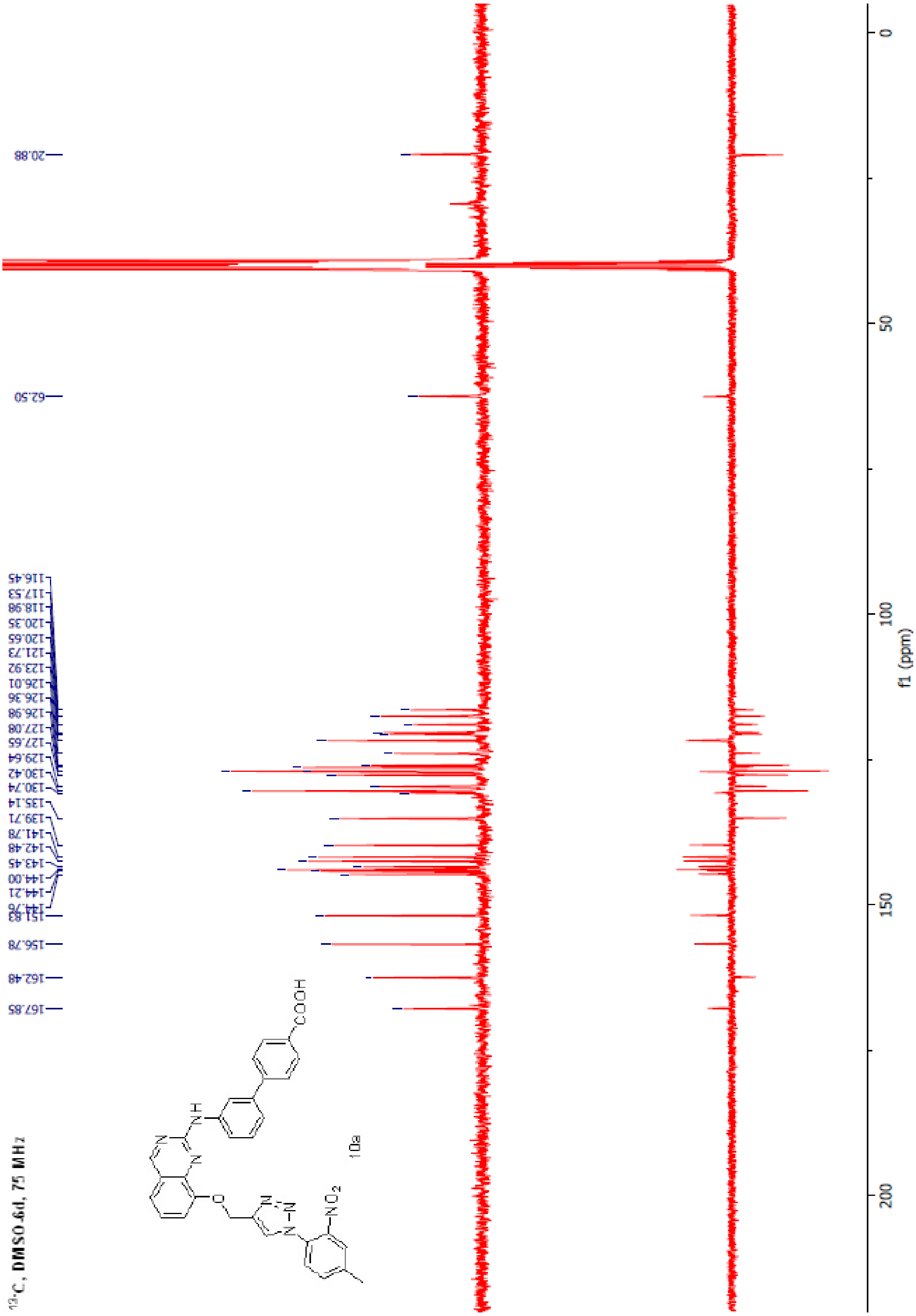

Figure S21: ^{13}C NMR spectra of **10a**

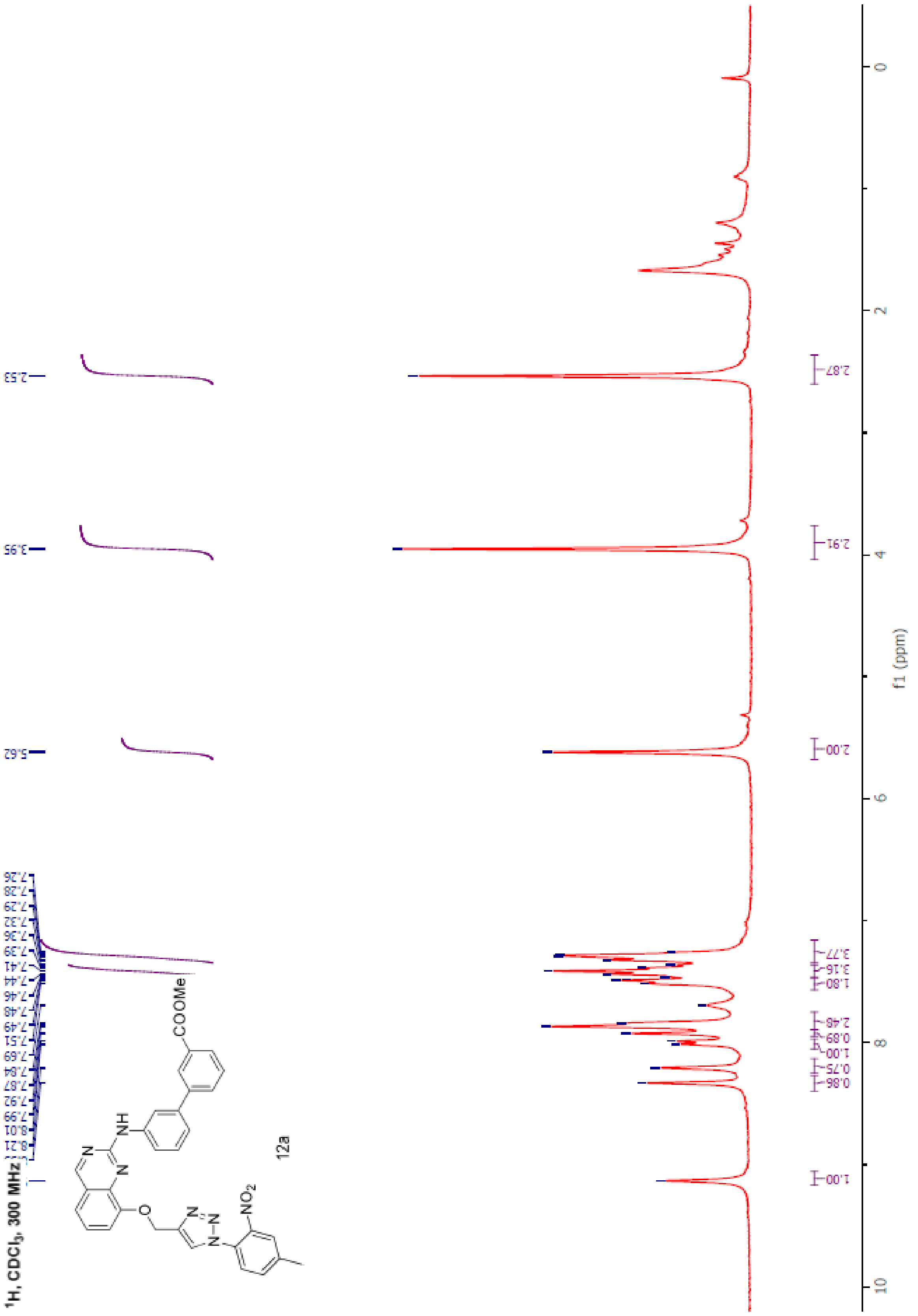

Figure S22: ^{1}H NMR spectrum of **12a**

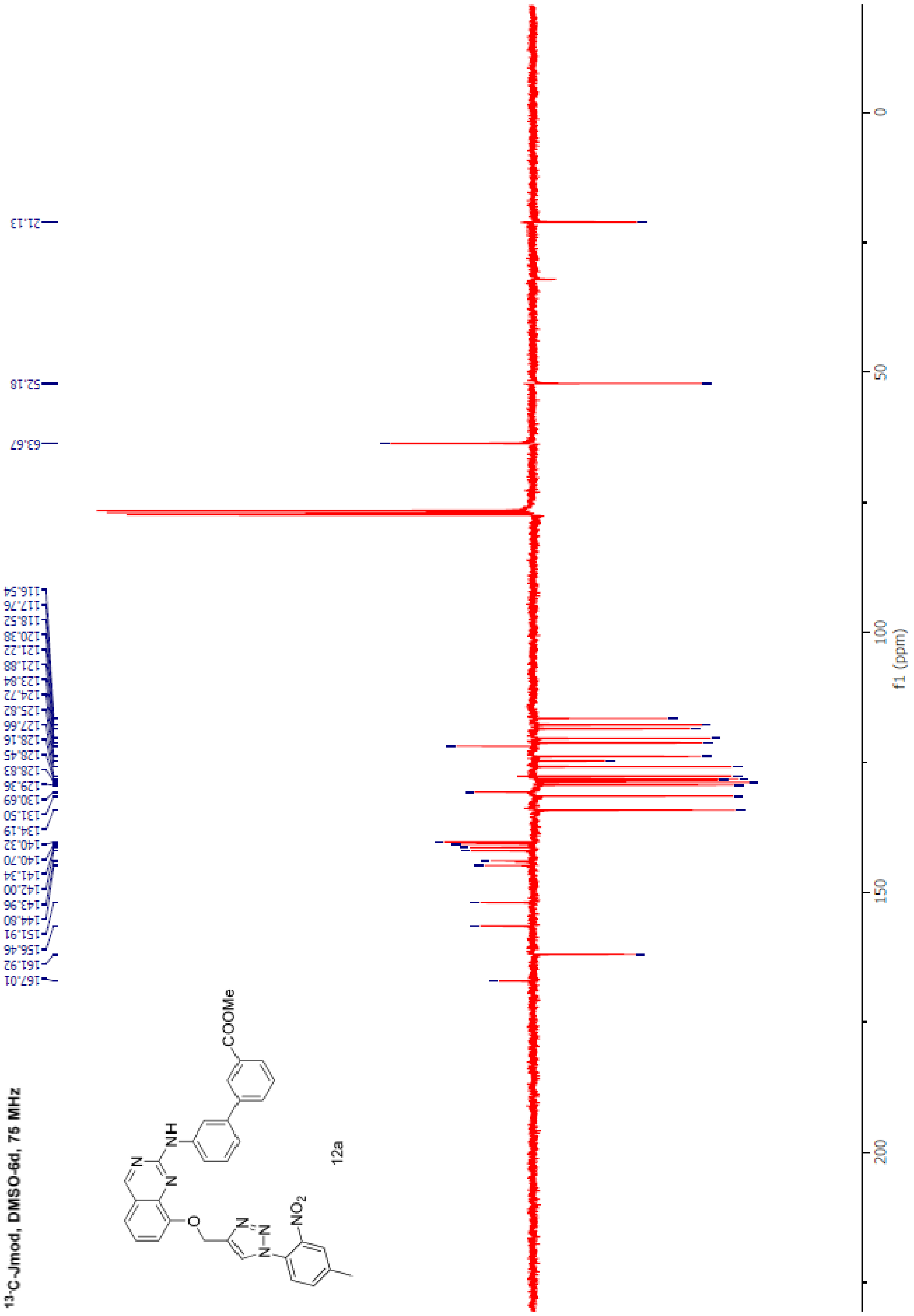

Figure S23: ^{13}C NMR spectrum of **12a**

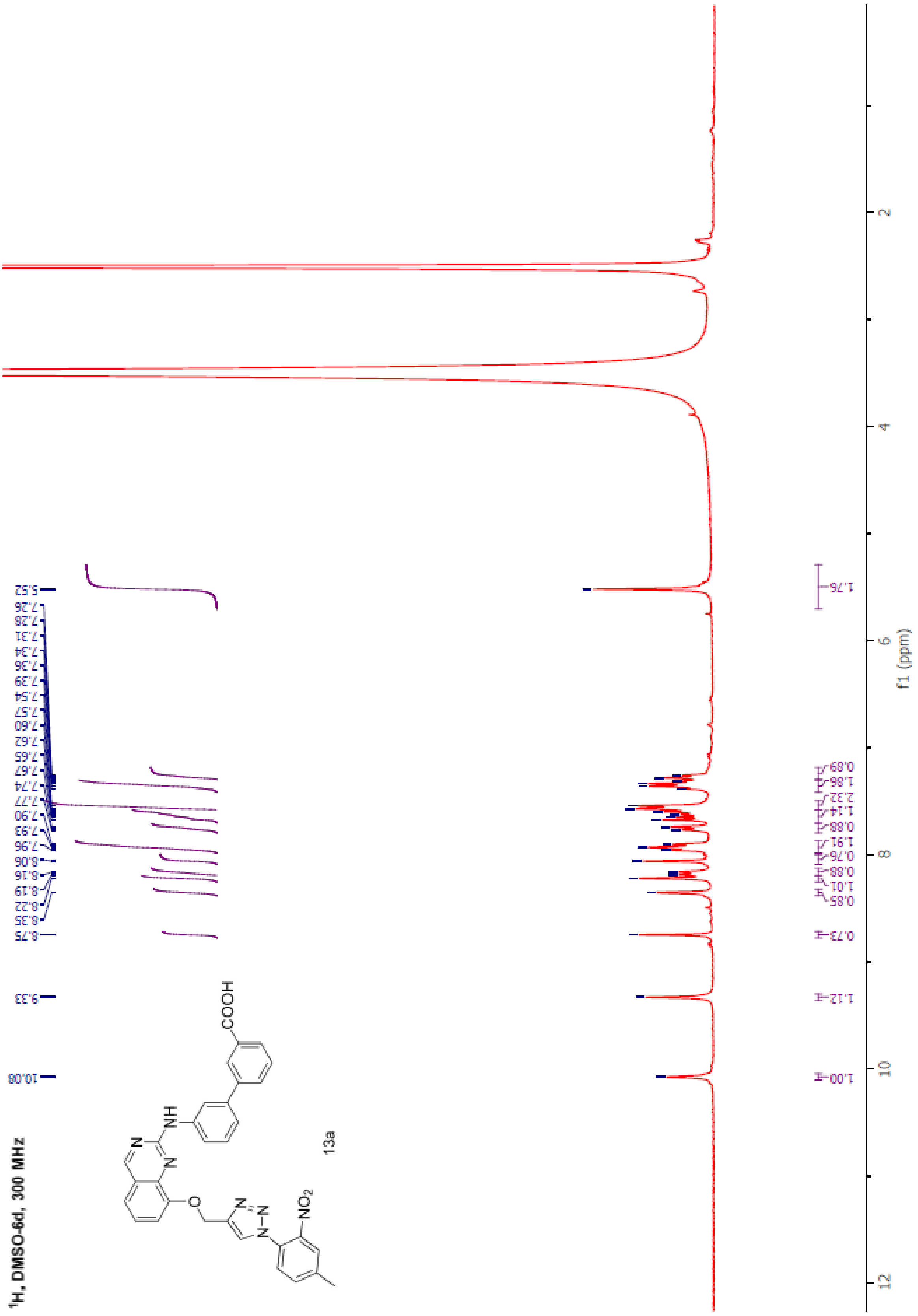

Figure S24: ^{1}H NMR spectrum of **13a**

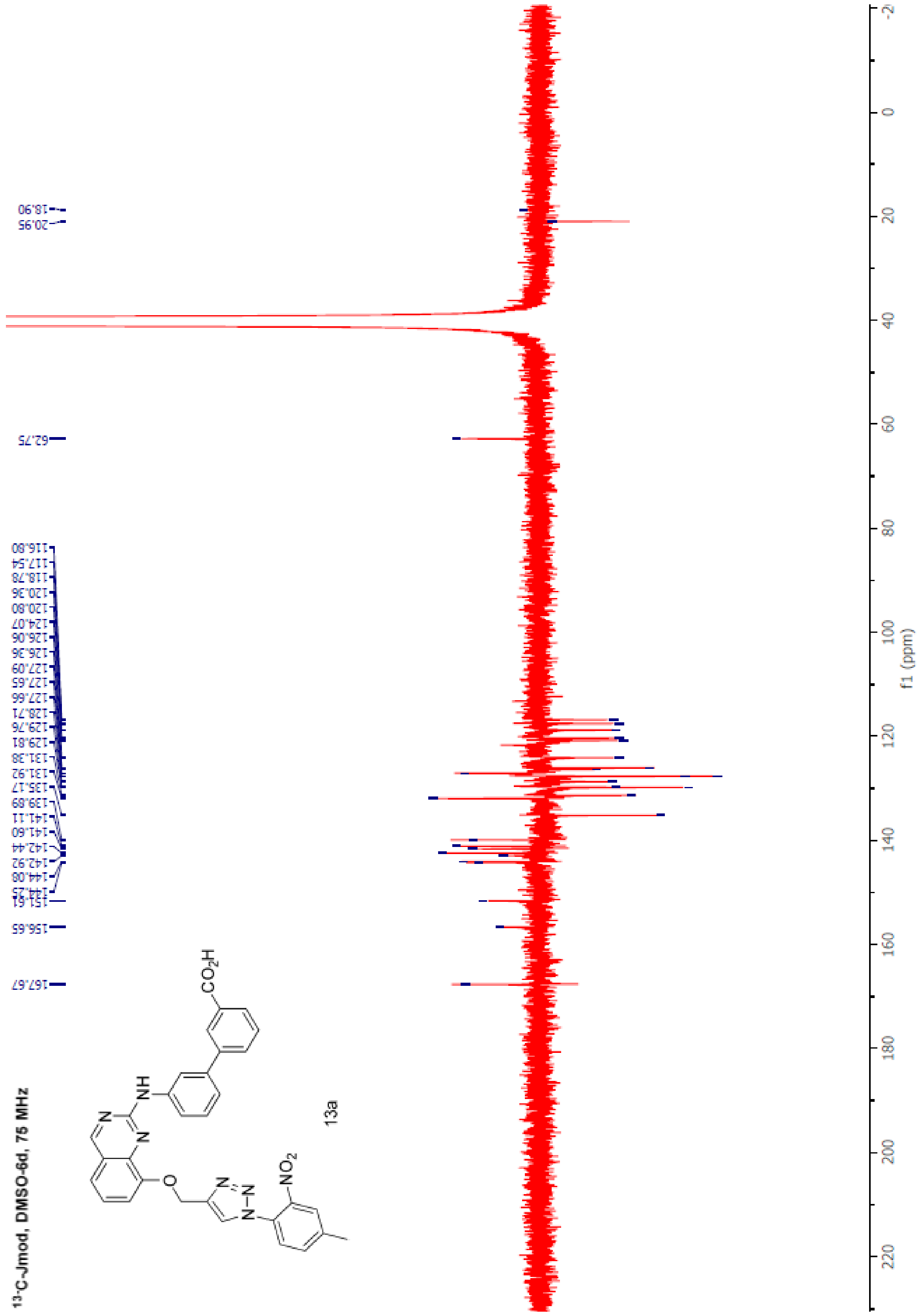

Figure S25: ^{13}C NMR spectrum of **13a**

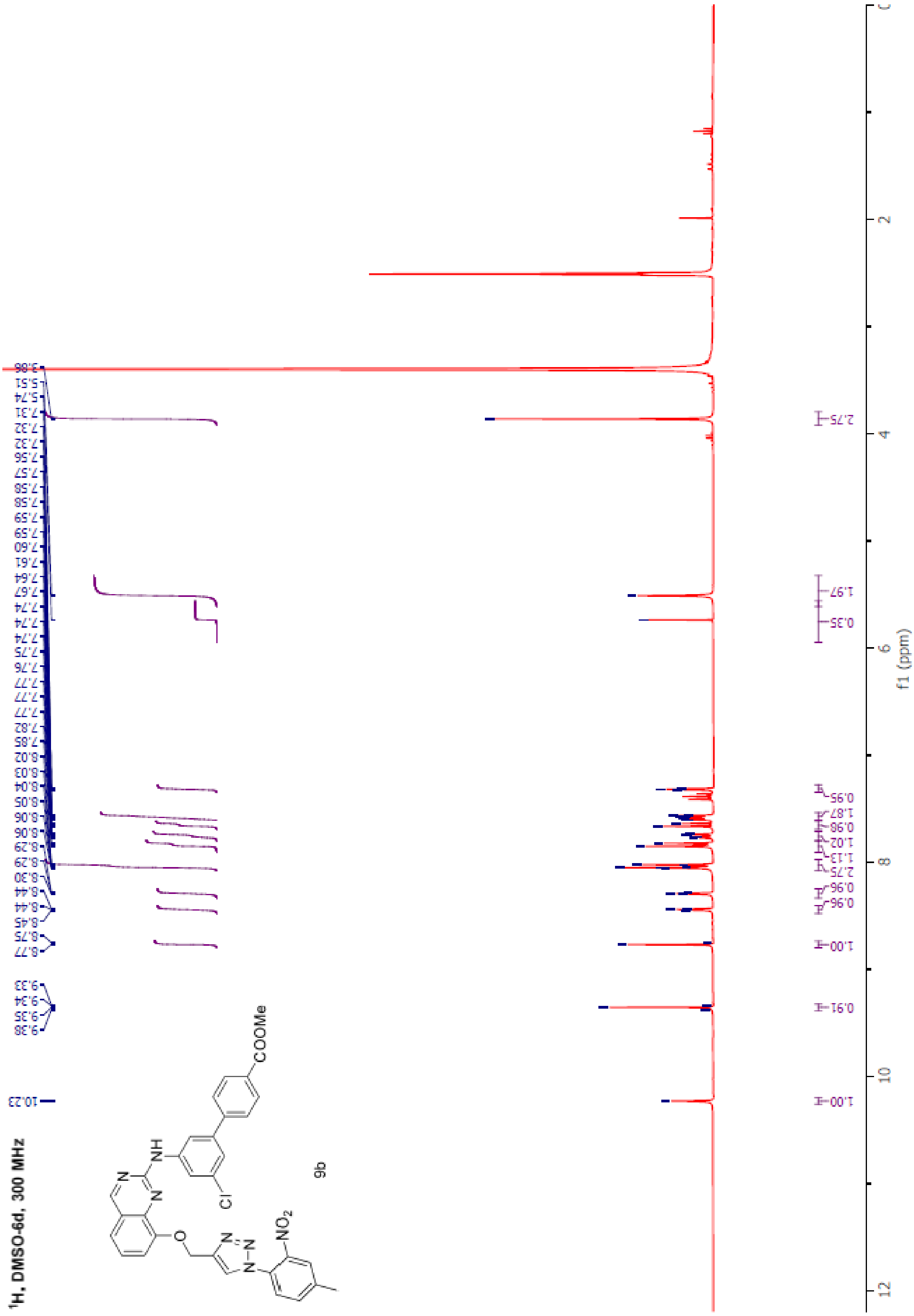

Figure S26: ^{1}H NMR spectrum of **9b**

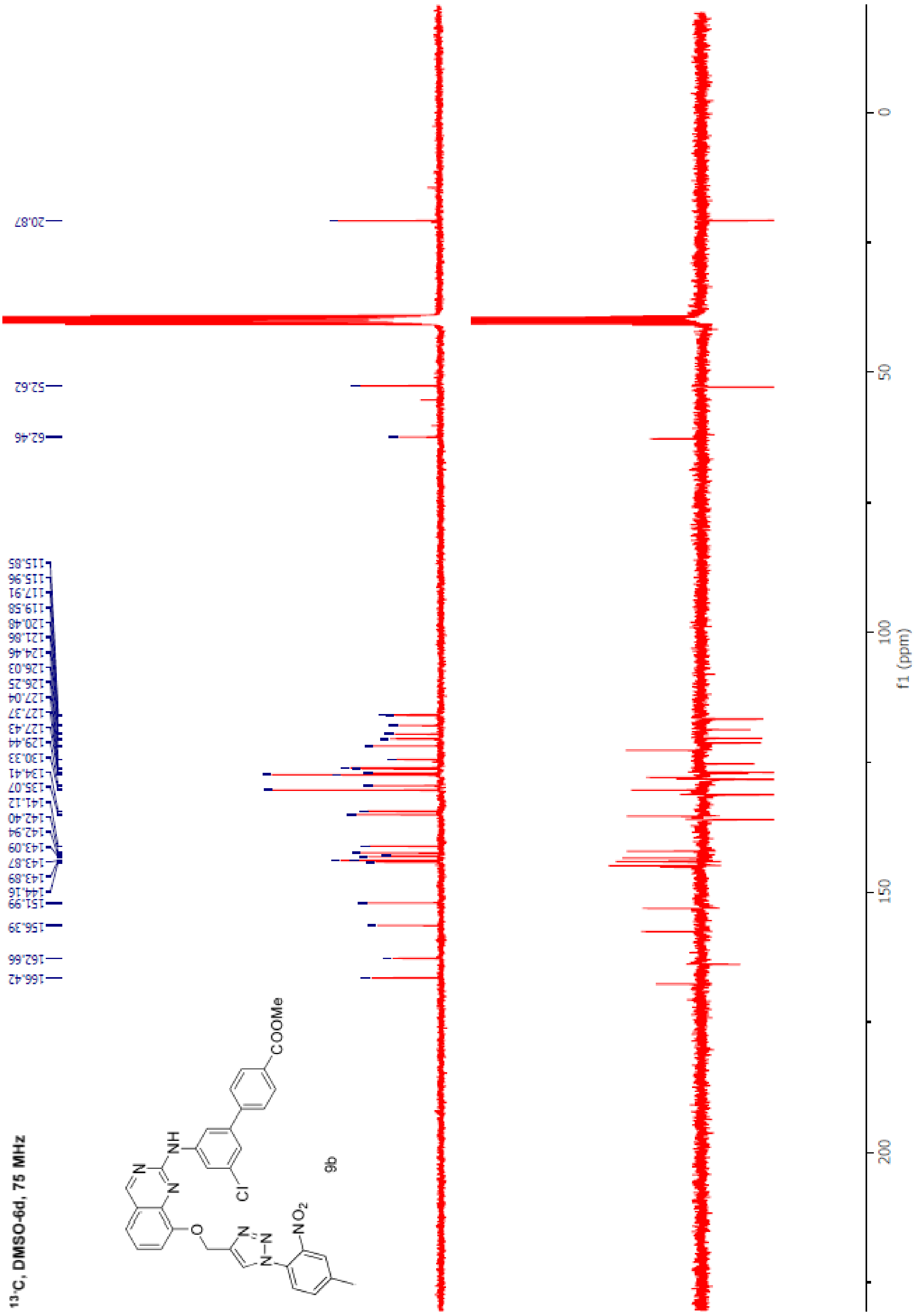

Figure S27: ^{13}C NMR spectra of **9b**

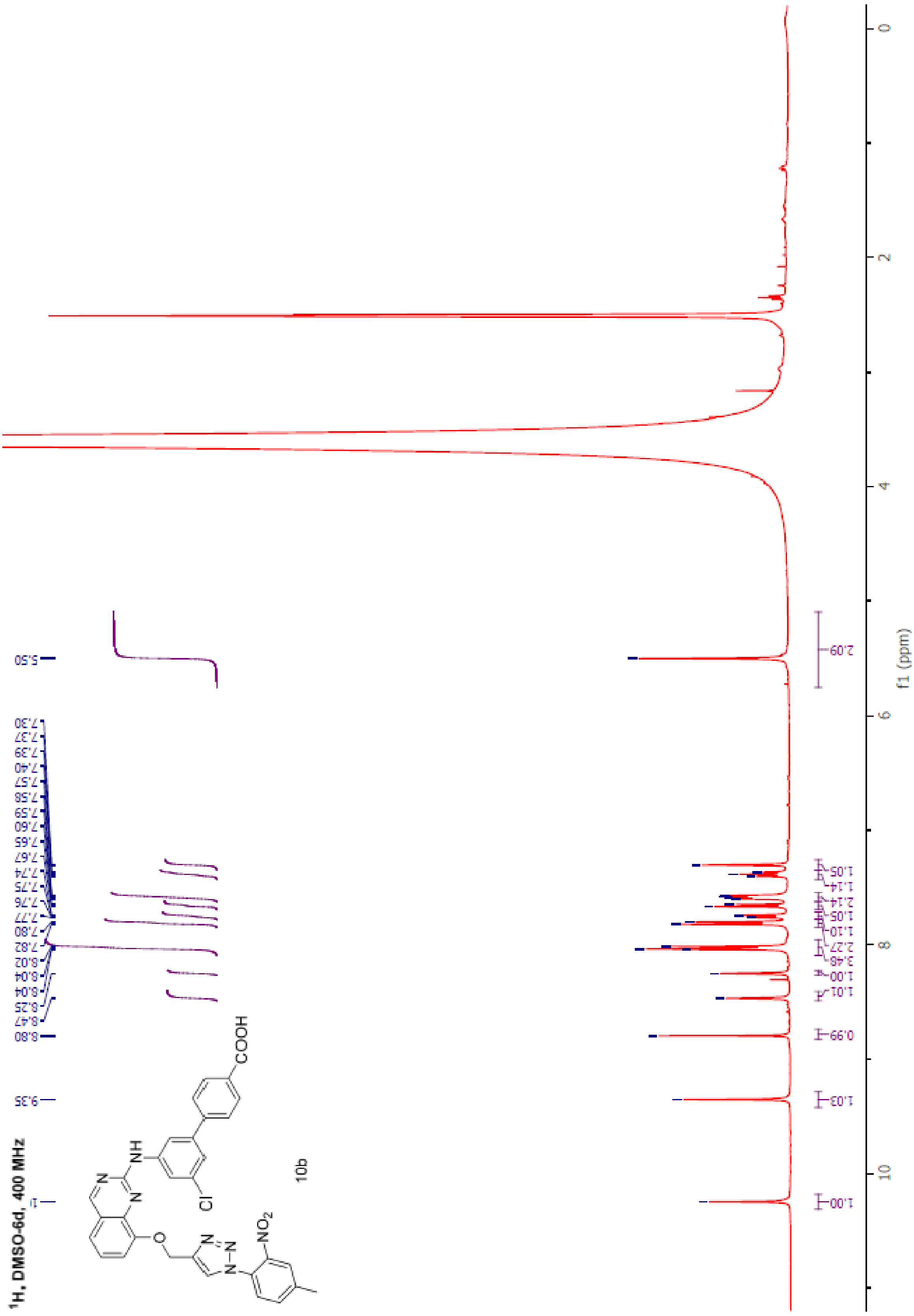

Figure S28: ^{1}H NMR spectrum of **10b**

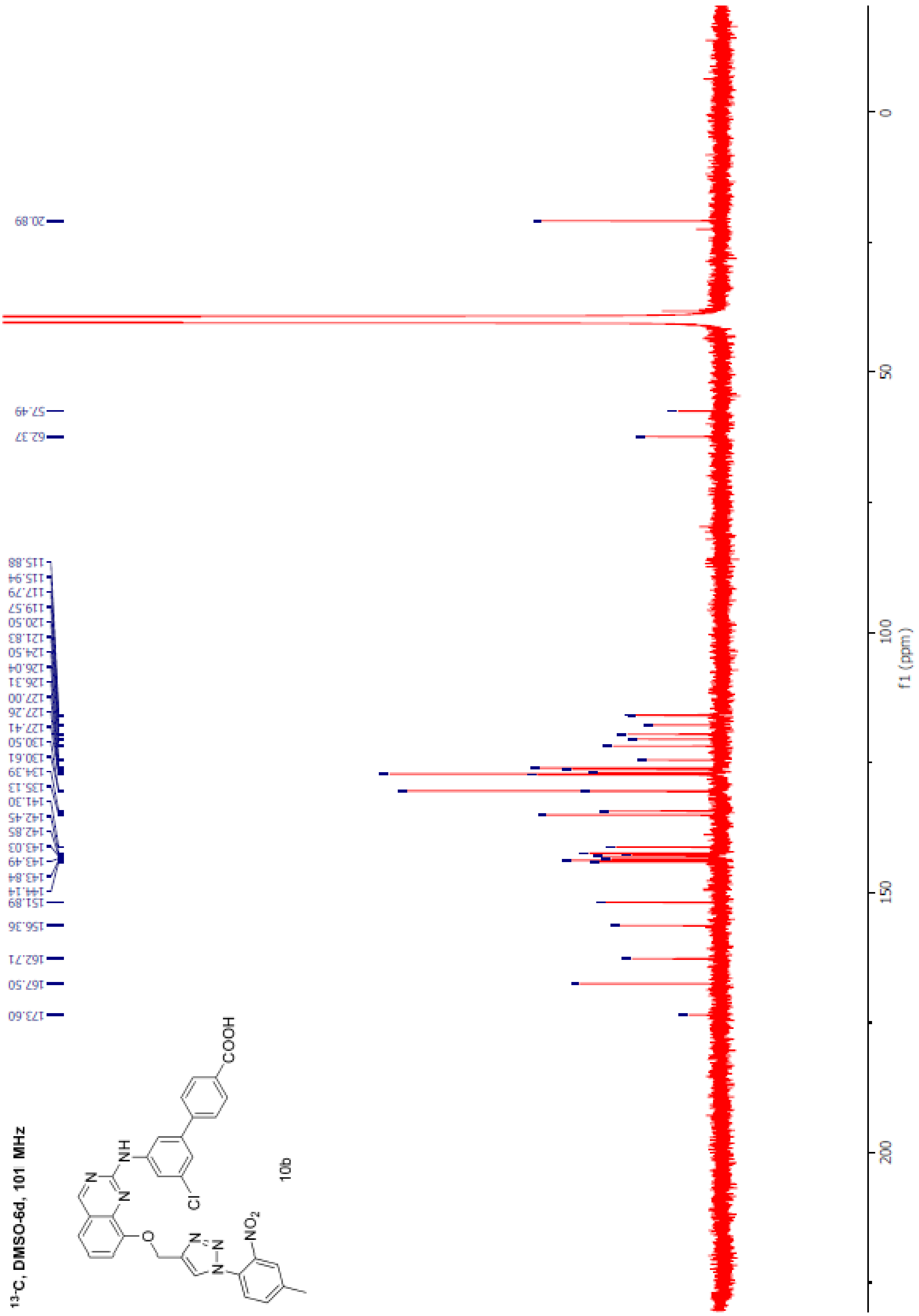

Figure S29: ^{13}C NMR spectrum of **10b**

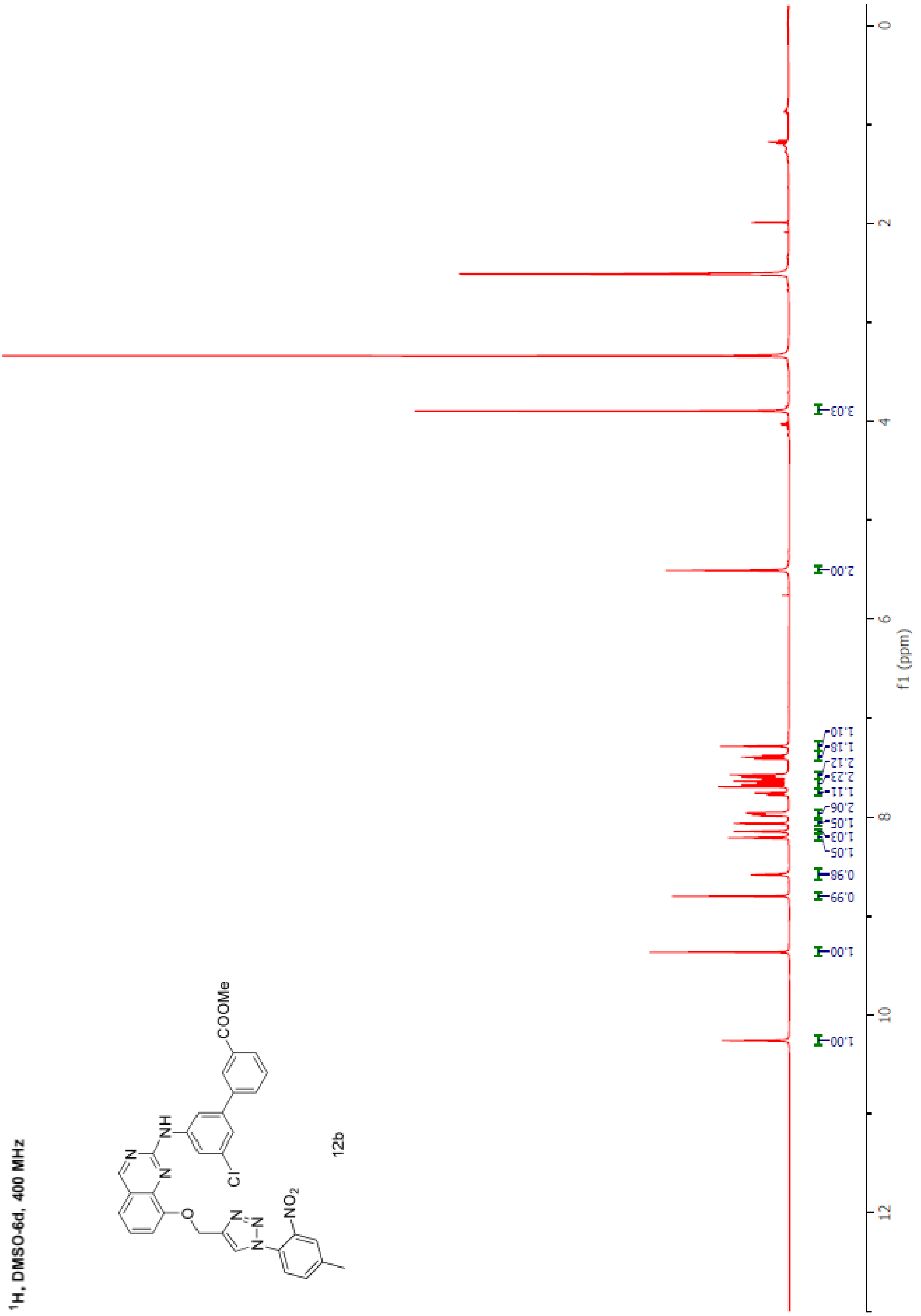

Figure S30: ^{1}H NMR spectrum of **12b**

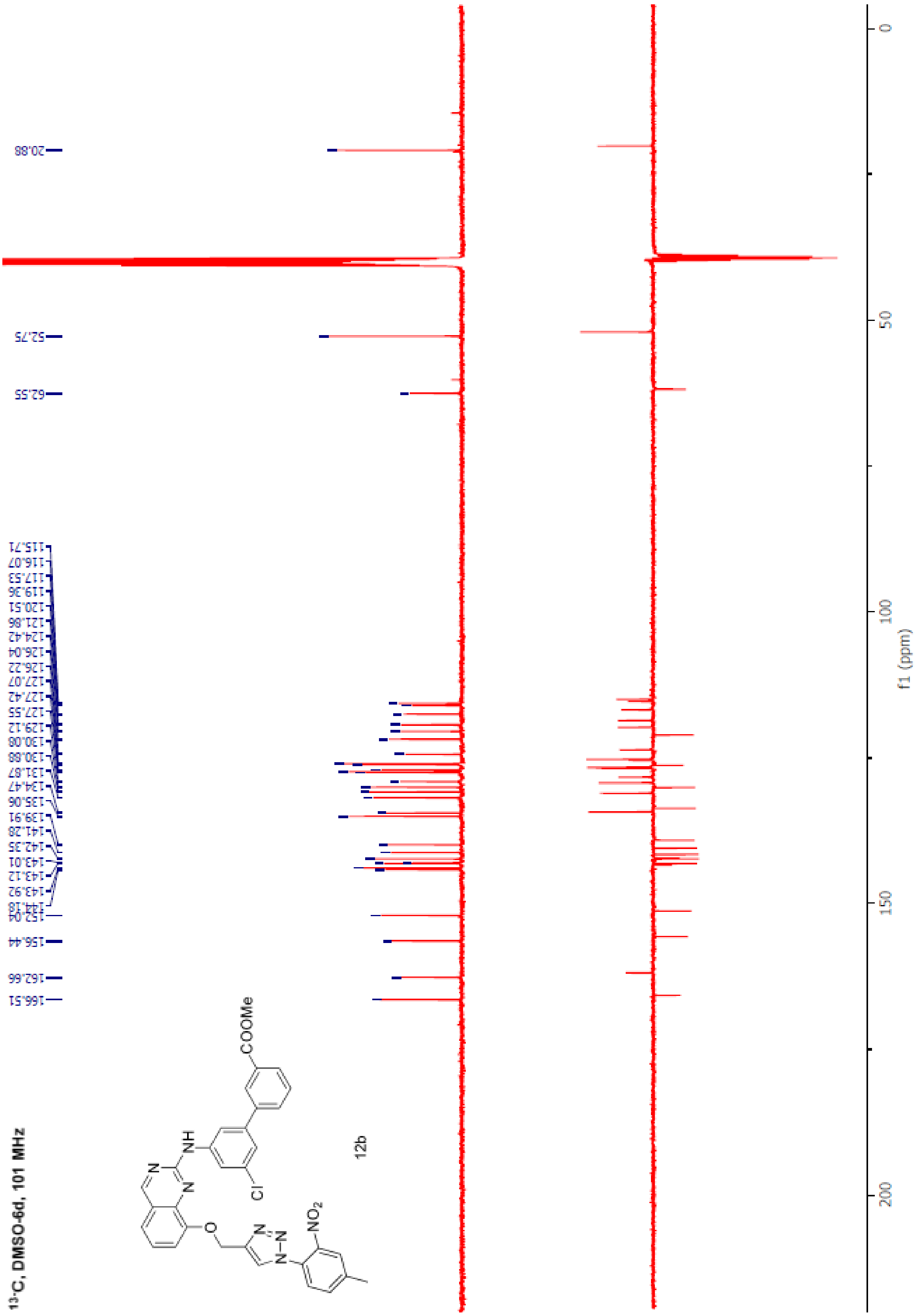

Figure S31: ^{13}C NMR spectra of **12b**

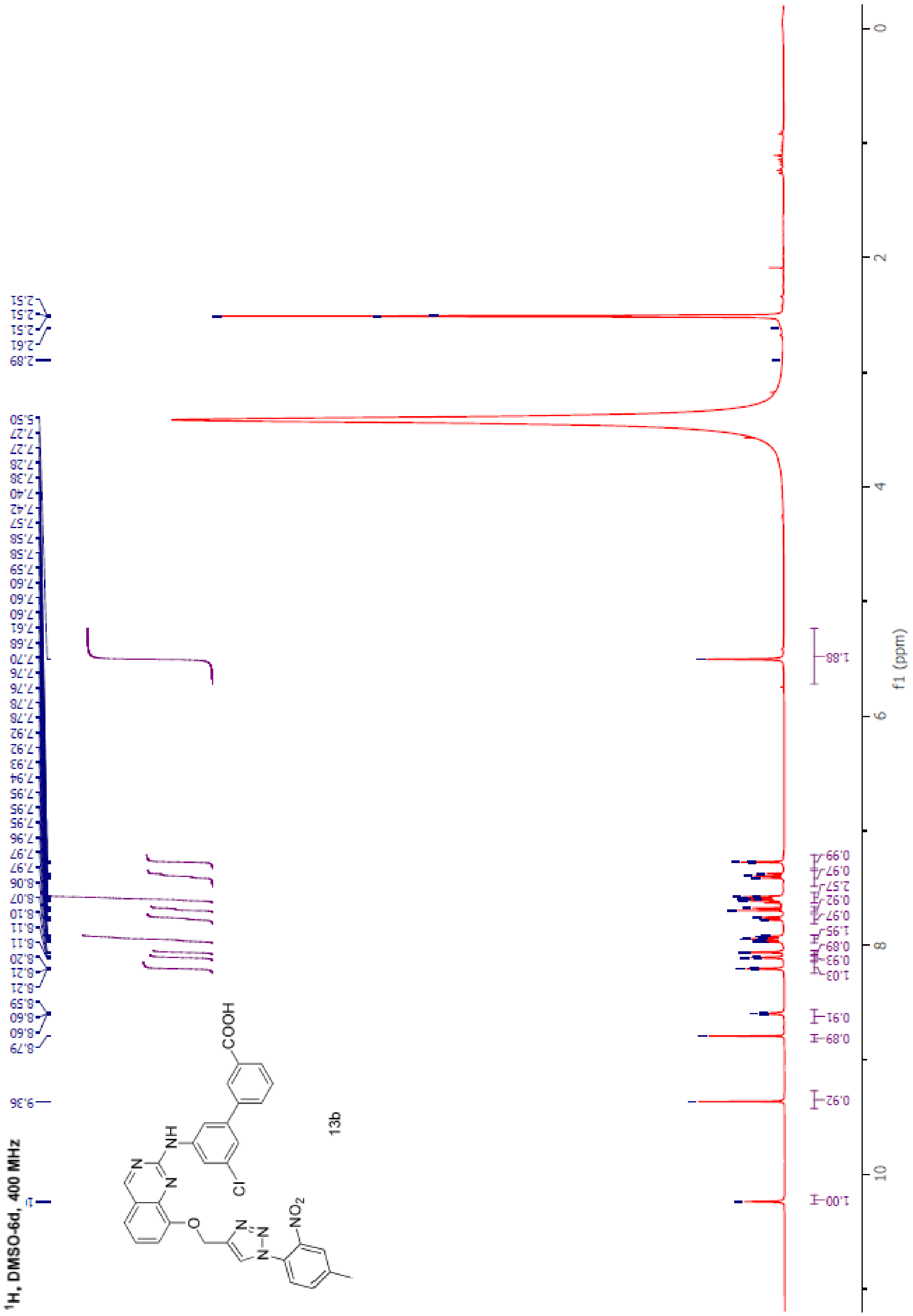

Figure S32: ^{1}H NMR spectrum of **13b**

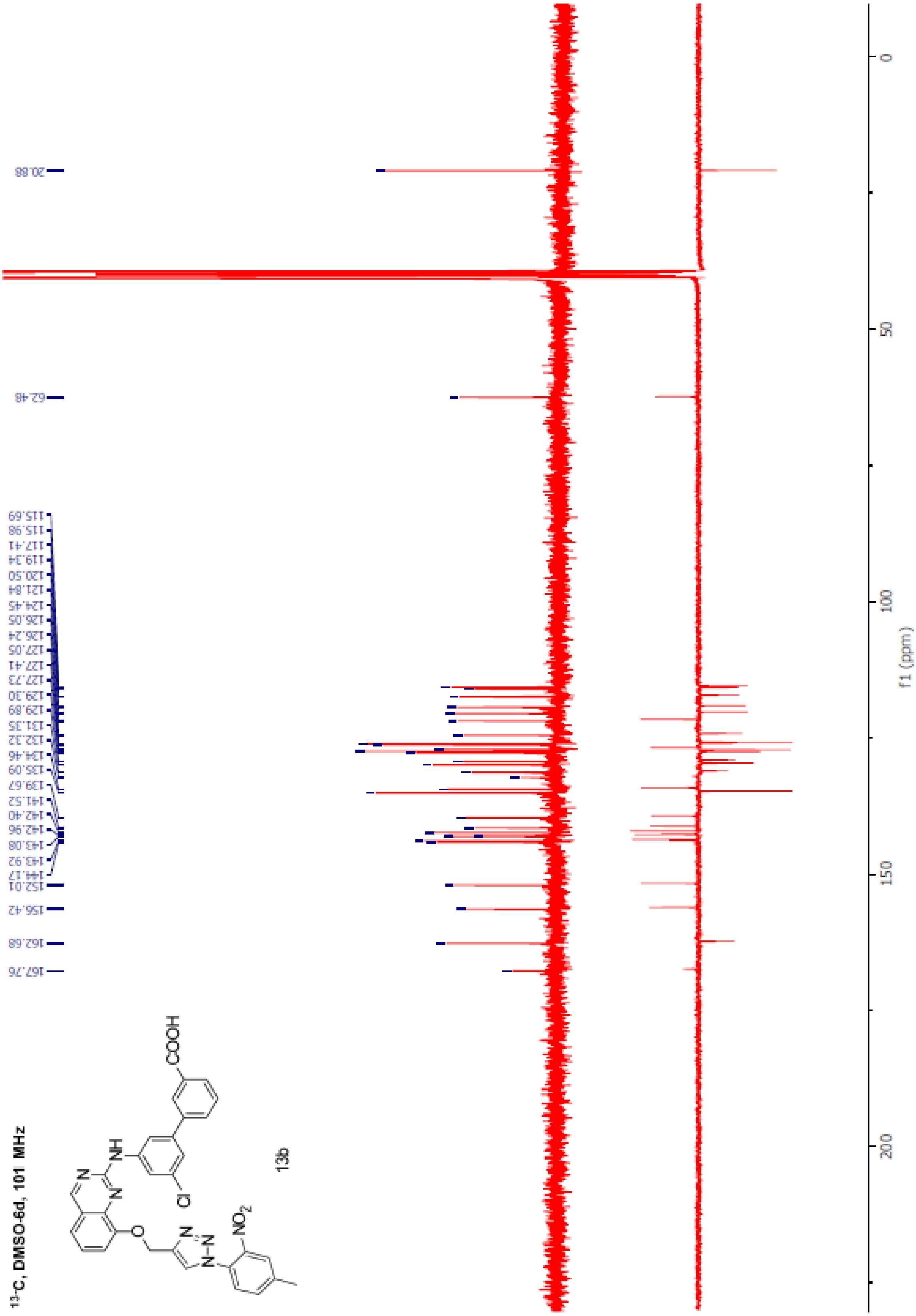

Figure S33: ^{13}C NMR spectrum of **13b**
